# Asymmetrically Functionalized Electron‐Deficient *π*‐Conjugated System for Printed Single‐Crystalline Organic Electronics

**DOI:** 10.1002/advs.202207440

**Published:** 2023-09-15

**Authors:** Craig P. Yu, Shohei Kumagai, Michitsuna Tsutsumi, Tadanori Kurosawa, Hiroyuki Ishii, Go Watanabe, Daisuke Hashizume, Hiroki Sugiura, Yukio Tani, Toshihiro Ise, Tetsuya Watanabe, Hiroyasu Sato, Jun Takeya, Toshihiro Okamoto

**Affiliations:** ^1^ Material Innovation Research Center (MIRC) and Department of Advanced Materials Science Graduate School of Frontier Sciences The University of Tokyo 5‐1‐5 Kashiwanoha Kashiwa Chiba 277‐8561 Japan; ^2^ Department of Applied Physics Faculty of Pure and Applied Sciences University of Tsukuba 1‐1‐1 Tennodai Tsukuba Ibaraki 305‐8573 Japan; ^3^ Department of Physics School of Science Kitasato University 1‐15‐1 Kitasato, Minami‐ku Sagamihara Kanagawa 252‐0373 Japan; ^4^ RIKEN Center for Emergent Matter Science (CEMS) 2‐1 Hirosawa Wako Saitama 351‐0198 Japan; ^5^ FUJIFILM Corp. 577 Ushijima, Kaisei‐machi Ashigarakami‐gun Kanagawa 258‐8577 Japan; ^6^ Rigaku Corp. 3‐9‐12 Matsubara‐cho Akishima Tokyo 196‐8666 Japan; ^7^ International Center for Materials Nanoarchitectonics (MANA) National Institute for Materials Science (NIMS) 1‐1 Namiki Tsukuba 205‐0044 Japan; ^8^ PRESTO, JST 4‐1‐8 Honcho Kawaguchi Saitama 332‐0012 Japan; ^9^ Department of Chemical Science and Engineering, School of Materials and Chemical Technology Tokyo Institute of Technology 4259‐G1‐7 Nagatsuta Midori‐ku Yokohama 226‐8502 Japan

**Keywords:** asymmetric n‐type organic semiconductors, large‐area single‐crystalline thin films, molecular design, nitrogen‐containing *π*‐electron systems, organic field‐effect transistors

## Abstract

Large‐area single‐crystalline thin films of n‐type organic semiconductors (OSCs) fabricated via solution‐processed techniques are urgently demanded for high‐end electronics. However, the lack of molecular designs that concomitantly offer excellent charge‐carrier transport, solution‐processability, and chemical/thermal robustness for n‐type OSCs limits the understanding of fundamental charge‐transport properties and impedes the realization of large‐area electronics. The benzo[*de*]isoquinolino[1,8‐*gh*]quinolinetetracarboxylic diimide (BQQDI) *π*‐electron system with phenethyl substituents (PhC_2_–BQQDI) demonstrates high electron mobility and robustness but its strong aggregation results in unsatisfactory solubility and solution‐processability. In this work, an asymmetric molecular design approach is reported that harnesses the favorable charge transport of PhC_2_–BQQDI, while introducing alkyl chains to improve the solubility and solution‐processability. An effective synthetic strategy is developed to obtain the target asymmetric BQQDI (PhC_2_–BQQDI–C*_n_*). Interestingly, linear alkyl chains of PhC_2_–BQQDI–C*_n_* (*n* = 5–7) exhibit an unusual molecular mimicry geometry with a *gauche* conformation and resilience to dynamic disorders. Asymmetric PhC_2_–BQQDI–C_5_ demonstrates excellent electron mobility and centimeter‐scale continuous single‐crystalline thin films, which are two orders of magnitude larger than that of PhC_2_–BQQDI, allowing for the investigation of electron transport anisotropy and applicable electronics.

## Graphical abstract

A series of asymmetric BQQDI (PhC_2_–BQQDI–C*_n_*, *n* = 5–7) n‐type organic semiconductors simultaneously showing excellent charge transport and solution‐processability are reported. Interestingly, their single‐crystal structures exhibit an unusual molecular mimicry geometry that provides resilience to dynamic disorders. Asymmetric PhC_2_–BQQDI–C*_n_* with the pentyl chain demonstrates high electron mobility in centimeter‐scale single‐crystalline thin films.

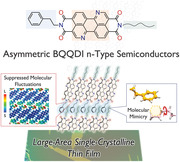

## 1 Introduction

Single‐crystalline organic semiconductors (OSCs)^[^ ^1^, ^2^, ^3^ ^]^ with continuous uniform domains offer direct access to their intrinsic charge transport due to the absence of grain boundaries, which is ideal for investigating fundamental charge‐transport properties and achieving high charge‐carrier mobility (*µ*) for practical applications. While conventional single‐crystalline OSCs are grown via sublimations in small scales that are difficult to handle and impractical to fabricate integrated circuits consisting numerous transistors,^[^ ^4^, ^5^, ^6^, ^7^ ^]^ recent advances in solution‐processed printing technologies enable the fabrication of single‐crystalline thin films up to wafer scales with thicknesses of around ten nanometers directly on device substrates.^[^ ^8^, ^9^, ^10^, ^11^ ^]^ These large‐area single‐crystalline OSCs may have more than one thousand transistors on a single uniformed surface.^[^ ^12^, ^13^, ^14^ ^]^ As a result, strain sensors^[^ ^15^ ^]^ and highly integrated circuits that combine both hole‐transporting p‐type and electron‐transporting n‐type OSCs are eminently anticipated.^[^ ^16^, ^17^, ^18^ ^]^ State‐of‐the‐art p‐type OSCs generally demonstrate the 2D herringbone assembly with effective intermolecular orbital overlaps,^[^ ^19^, ^20^, ^21^ ^]^ leading to excellent charge transport and high *µ* over 10 cm^2^ V^−1^ s^−1^.^[^ ^21^, ^22^ ^]^ The incorporation of alkyl chains gives p‐type OSCs high solubility in common organic solvents and the formation of solution‐processed large‐area single‐crystalline thin films,^[^ ^8^, ^9^, ^10^, ^11^ ^]^ leading to high‐end electronics. Furthermore, the observation of 2D hole gas and metal‐insulator transition in defect‐free solution‐processed single‐crystalline thin‐film p‐type OSC may signify the advancement of OSCs into the realm of quantum electronics.^[^ ^23^ ^]^

The n‐type OSCs, on the other hand, lag behind their p‐type counterparts both in terms of *µ* and solution‐processability. Since n‐type OSCs require deep‐lying lowest unoccupied molecular orbital (LUMO) energy levels for charge injections and air‐stable electron transports,^[^ ^24^, ^25^, ^26^, ^27^ ^]^ the incorporation of polar electron‐deficient moieties generally lead to anisotropic 1D molecular assemblies with ineffective intermolecular orbital overlaps and poor charge transports. In contrast to the high‐performance p‐type OSCs that show excellent solution‐processability, the 1D molecular assemblies of n‐type OSCs also likely limit the formation of 2D large‐area single‐crystalline thin films, which impedes the realization of high‐speed and low‐cost organic‐based integrated circuits with low power consumptions.

**Figure 1:**
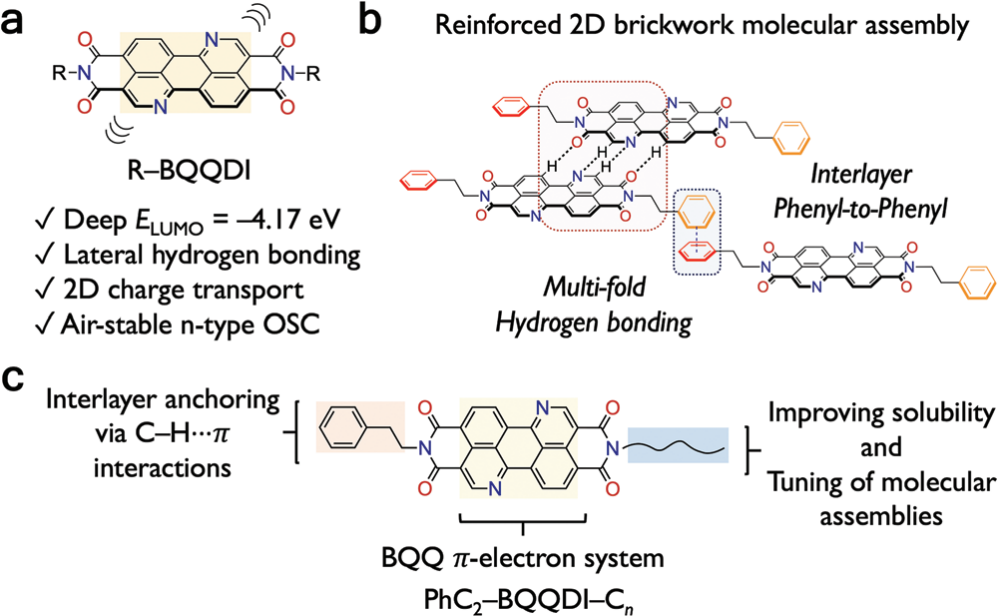
a) Molecular structure and features of R–BQQDI. b) Molecular assembly PhC_2_–BQQDI, c) design of asymmetric PhC_2_–BQQDI–C*_n_* (C*_n_*: linear alkyl chains, *n*‐C*_n_* H_2_ *_n_*_+1_).

The electron‐deficient benzo[*de*]isoquinolino[1,8‐*gh*]quinolinetetracarboxylic diimide (BQQDI) is reported as a *π*‐electron system for n‐type OSCs (**Figure** **1**a).^[^ ^28^, ^29^, ^30^, ^31^ ^]^ Though BQQDI bares structural similarity with the well‐studied perylene diimide (PDI),^[^ ^32^, ^33^, ^34^, ^35^ ^]^ the incorporated electronegative nitrogen atoms in BQQDI contribute to a deep‐lying LUMO level (Figure 1a), offering air‐stable electron transports. The symmetric phenethyl‐substituted–BQQDI (PhC_2_–BQQDI) shows multi‐fold intermolecular hydrogen‐bonding‐like C−H···N and C−H···O interactions between BQQDI *π*‐electron cores, and C–H···*π* interactions between phenyl groups, which lead to a 2D brickwork assembly with excellent charge transport and resilience to dynamic disorders (Figure 1b). The single‐crystalline thin film of PhC_2_–BQQDI exhibits a high *µ* with band‐like charge transport. However, the high‐performance PhC_2_–BQQDI shows issues of low solubility arising from strong aggregation due to rigid substituents, which could be the primary detrimental factor for large‐area processability.

From the examples of p‐type OSCs, it is apparent that the alkyl chains are essential for the formation of large‐area solution‐processed single‐crystalline thin films due to enhanced layered ordering.^[^ ^10^, ^13^, ^36^, ^37^ ^]^ Though, the phenethyl substituent of PhC_2_–BQQDI is extremely effective in reinforcing the intermolecular orbital overlaps and providing resilience to dynamic disorders. In this work, we are interested in the molecular design that harnesses the excellent charge transport and resilience to dynamic disorder of PhC_2_–BQQDI, while achieving high solubility and solution‐processability. Thus, we envisage a design strategy that combines the molecular features of the phenethyl and alkyl substituents in one asymmetric BQQDI molecule (PhC_2_–BQQDI–C*_n_*) (Figure 1c). The asymmetric design approach has been investigated on various thienoacene p‐type OSCs, which offers exceptional solution‐processability,^[^ ^13^, ^38^ ^]^ although this strategy has not been applied for n‐type large‐area single‐crystalline thin films OSCs. While the current asymmetric design may primarily improve the solubility, we expect tuning of inter‐substituent interactions between the adjacent 2D assemblies, which can lead to large‐area 2D single‐crystalline thin films. However, obtaining asymmetric diimide‐containing *π*‐electron systems remains a synthetic challenge. The synthesis of asymmetric PDI derivatives generally involves sequential imidizations that result in the desired monoimidized product, along with unreacted and difunctionalized species, which are attributed to the high reactivity of primary alkylamines.^[^ ^39^, ^40^, ^41^ ^]^ Owing to the relatively low solubility of PDI, purification of the desired asymmetric compound can be difficult, leading to poor yields and low purity.

Herein, we developed an effective and selective synthetic method to obtain the target asymmetric PhC_2_–BQQDI–C*_n_* (*n* = 5, 6 and 7), where the favorable phenethyl sidechain is preserved on one side, and flexible alkyl chains are introduced the other side of BQQDI. The introduction of alkyl chains in current asymmetric PhC_2_–BQQDI–C*_n_* derivatives show higher solubility than that of PhC_2_–BQQDI by one order of magnitude, which significantly improves the solution‐processability for large‐area thin‐film fabrications. We report that the alkyl chains of PhC_2_–BQQDI–C*_n_* mimic the overall shape of the surrounding phenyl groups by adopting the *gauche* conformation that is stabilized by multiple C−—H···*π* interactions and demonstrate a molecular mimicry assembly. In particular, the PhC_2_–BQQDI–C_5_ derivative shows air‐stable and high n‐type OSC performances in solution‐processed organic field‐effect transistors (OFETs), and centimeter‐scale single‐crystalline thin films are obtained, which allows us to study the directionality of *µ* and compare theoretical and experimental evaluations.

## 2 Results and Discussion

### 2.1 Synthesis

**Scheme 1:**
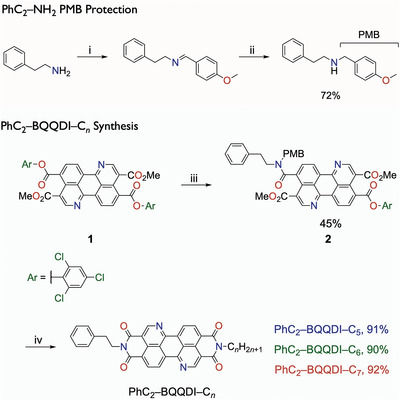
Preparation of PMB‐protected phenethylamine, and synthesis of PhC_2_–BQQDI–C*_n_* (*n =* 5, 6 and 7). Reagents and conditions: i) 4‐methoxybenzaldehyde, magnesium sulfate, dichloromethane, r.t., 2 h. ii) Sodium borohydride, dichloromethane/methanol, 0 ˚C to r.t., 2 h. iii) PMB‐protected phenethylamine, *o*‐DCB, 180 ˚C, 40 min. iv) 1) Alkylamine, *o*‐DCB, 150 ˚C, 1 h, 2) triflic acid, 150 ˚C, 3 h.

Although nature synthesizes asymmetric biomolecules with marvelous efficiencies, it is a challenge for synthetic chemists to prepare asymmetric compounds due to poor selectivity and low yields.^[^ ^42^ ^]^ One of the main reasons leading to synthetic issues of asymmetric PDI is the high reactivity of primary alkylamines. Thus, we speculate a more rational and selective method needs to be developed to prepare the target asymmetric BQQDI derivatives. We lower the reactivity of the phenethylamine by functionalizing it with the *p*‐methoxybenzyl (PMB) group (**Scheme** **1**), which is a heat‐stable protecting group that can endure imidization reactions at elevated temperatures and can be eventually removed by acid.

We began our synthesis from the previously reported trichlorophenyl ester‐containing compound **1**,^[^ ^28^ ^]^ as the versatile electron‐deficient ester can be readily displaced by alkylamines and purified by column chromatography. The PMB‐protected amine was then reacted with compound **1** in refluxed *o*‐dichlorobenzene (*o*‐DCB) for 40 min to give intermediate **2** in 45% yield. Although the first reaction generated the desired and difunctionalized products, as well as unreacted compound **1** indicated by high‐performance liquid chromatography, compound **2** was readily isolated by column chromatography. From the key precursor **2**, we carried out a highly selective one‐pot synthesis to furnish a series of PhC_2_–BQQDI–C*_n_* from intermediate **2**, as PMB removal and ring‐closing steps were both facilitated by TfOH, and the one‐pot synthesis resulted in excellent yields of 90–92%.

### 2.2 Physicochemical Properties

The thermal stability of PhC_2_–BQQDI–C*_n_* derivatives was evaluated by thermalgravimetric‐differential thermal analysis, and the crystal phase stability/transition was measured by differential scanning calorimetry (DSC). All PhC_2_–BQQDI–C*_n_* derivatives showed excellent thermal stability with 5% weight loss temperatures and decomposition temperatures above 370 and 380 ˚C, respectively (Figure S6, Supporting Information). DSC measurements indicated no apparent phase transitions of PhC_2_–BQQDI–C*_n_* up to 350 ˚C (Figure S7, Supporting Information). All PhC_2_–BQQDI–C*_n_* derivatives exhibited completely reversible reduction waves in cyclic voltammetry (CV) measurements in solution (Figure S8, Supporting Information). The length of alkyl chains did not impose noticeable effects in electrochemical properties, as all derivatives showed first half‐wave reduction potentials of −0.68 V that corresponded to *E*_LUMO_ = −4.12 eV, and the second reduction potentials of −1.0 V appeared to be reversible and electrochemically stable. The electrochemical properties of PhC_2_–BQQDI–C*_n_* indicate a deep‐lying LUMO level that is suitable for air‐stable n‐channel OFET operations.^[^ ^25^ ^]^ Owing to the electron‐deficient nature of the BQQDI *π*‐electron core, the deep highest occupied molecular orbital (HOMO) energy levels of PhC_2_–BQQDI–C*_n_* derivatives could not be obtained via CV measurements. We estimated the optical HOMO–LUMO gap of PhC_2_–BQQDI–C*_n_* (2.27 eV) from their solution‐state UV–vis spectra (Figure S9, Supporting Information), giving an *E*_HOMO_ of −6.39 eV. Solid‐state optical gaps of BQQDI derivatives are estimated from the Tauc plot, which are in the range of 2.03–2.07 eV (Figure S10 and Table S1, Supporting Information).

### 2.3 Molecular Assemblies and Charge Transport

**Figure 2:**
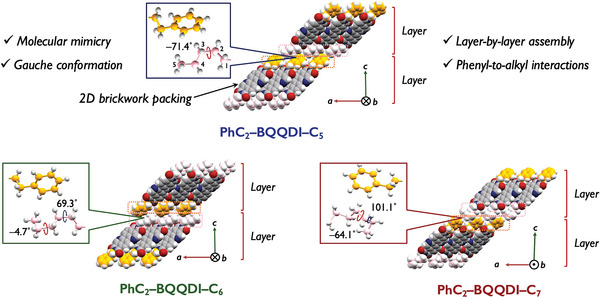
Packing structures and interlayer interactions of PhC_2_–BQQDI–C*_n_* (one of the conformers of PhC_2_–BQQDI–C_7_ is shown).

We examined the plate‐like single crystals of PhC_2_–BQQDI–C*_n_* grown by slow cooling in solutions (Table S2, Supporting Information). PhC_2_–BQQDI–C*_n_* derivatives form the 2D brickwork packing motif with *π–π* stacking and transverse hydrogen‐bonding‐like interactions (**Figure** **2**). The asymmetric molecules did not form the phenyl‐to‐phenyl interlayer interactions shown in PhC_2_–BQQDI, instead, the alkyl chains interact with phenyl groups in the adjacent layer (Figure 2). An intriguing finding of PhC_2_–BQQDI–C*_n_* derivatives is their molecular mimicry assemblies by the alkyl chain conformations. Instead of the linear *anti* conformation, PhC_2_–BQQDI–C_5_ shows a *gauche* conformation at the C2–C3 bond with a torsion angle of −71.4˚, and PhC_2_–BQQDI–C_6_ exhibits *gauche* conformations at C2–C3, and C4–C5 bonds with torsion angles of 69.3˚ and −4.8˚, respectively, while the hexyl chains show thermal disordering as revealed by molecular dynamics (MD) simulation (vide infra) (Figure S16, Supporting Information). By further extending the alkyl chain to *n* = 7, the single‐crystal structure exhibits static (site‐occupancy) disordering of the heptyl chains, though the molecular mimicry is still present (Figure 2).

The stabilization of the molecular mimicry is arguably attributed to the steric effect from the surrounding phenyl groups, and the large energy barrier between *anti* and *gauche* rotamers may disfavor isomerization at room temperature and provides resilience to molecular fluctuations. Such a stabilization of the *gauche* conformation of alkyl chains are also observed in several host‐guest systems^[^ ^43^ ^]^ and membrane protein structures.^[^ ^44^ ^]^ The powders of PhC_2_–BQQDI–C_5_ are further subjected to temperature‐variant powder X‐ray diffractions (PXRD) at SPring‐8 RIKEN Materials Science Beamline (BL44B2),^[^ ^45^, ^46^ ^]^ where the PXRD pattern is consistent with the single‐crystal structure, and consistent diffraction patterns are observed up to 200 ˚C with no apparent phase transitions, indicating that the intriguing molecular assembly is persistent (Figure S12, Supporting Information). The current findings of the molecular mimicry of is unique among OSCs including the asymmetric benzothieno[3,2‐*b*][1]benzothiophene (Ph–BTBT–C*_n_*) derivatives,^[^ ^47^ ^]^ where alkyl substituents are found to adopt the expected linear *anti* conformations. The geometric similarity between phenethyl and alkyl chains of PhC_2_–BQQDI–C*_n_* derivatives may allow the formation of molecular mimicry conformation.

**Figure 3:**
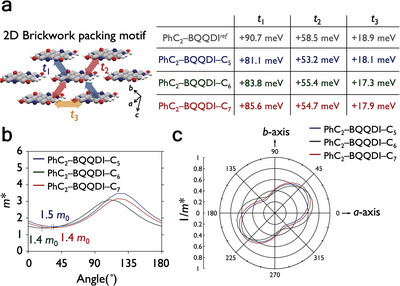
a) Transfer integral in the 2D brickwork packing motif calculated at the PBEPBE/6‐31G(d) level of theory. b) Effective mass (in units of *m*_0_, the rest mass of an electron). c) Angle‐dependent inversed effective mass (relative to crystallography axes) by the tight‐binding approximation of PhC_2_–BQQDI–C*_n_*.

Besides the intriguing interlayer assemblies, we investigated the charge‐transport capabilities of PhC_2_–BQQDI–C*_n_*. Multi‐fold hydrogen‐bonding‐like interactions are observed between the BQQDI *π*‐electron cores in PhC_2_–BQQDI–C*_n_* crystals, and four other *π–π* interactions are present in the 2D brickwork packing motif. To quantify the degree of LUMO overlap within the brickwork motif of PhC_2_–BQQDI–C*_n_*, transfer integral (*t*) values (*t*_1_, *t*_2_ and *t*_3_) were calculated at the PBEPBE/6‐31G(d) level (**Figure** **3**a). The *π*–*π* interactions of PhC_2_–BQQDI–C*_n_* exhibit large *t*_1_ and *t*_2_ values, with *t*_1_ values being larger than *t*_2_, suggesting anisotropic charge transport. The transverse directions also result in effective LUMO overlaps with positive *t*_3_ values ranging from +17.3 to +18.1 meV. With the tight‐binding approximation, effective mass of electron carriers (m*) of PhC_2_–BQQDI–C*_n_* are estimated from their LUMO band dispersions. By plotting the inversed m* with respect to the crystallographic axes, the anisotropic charge transport of PhC_2_–BQQDI–C*_n_* derivatives is clearly visualized by the peanut‐like shapes (Figure 3b,c). According to OSCs adopting the band‐like charge transport, the *µ* is described by the following equation, where *q* is the elemental charge and *t* is the relaxation time.^[^ ^21^ ^]^

(1) $$\mu =q\frac{\tau }{m^{*}}$$

Nevertheless, the minimum m* of PhC_2_–BQQDI–C*_n_* are estimated to be 1.4–1.5, which are comparable to that of PhC_2_–BQQDI (1.3) and promising for achieving high *µ*_e_ in OFETs.

### 2.4 Molecular Fluctuations and Dynamic Disorders

**Figure 4:**
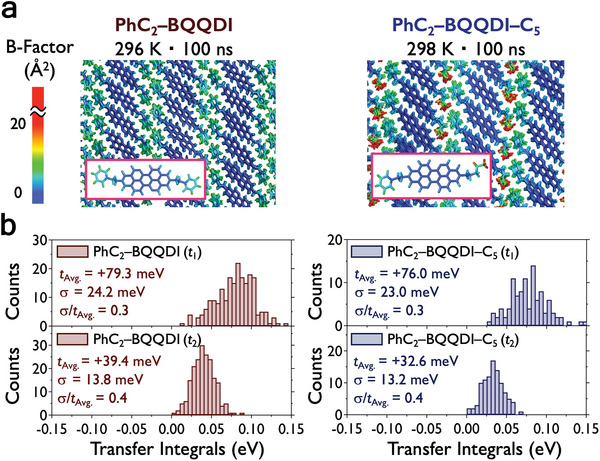
a) Color‐coded B‐factor (Å^2^) distribution of PhC_2_–BQQDI and PhC_2_–BQQDI–C*_n_* obtained from trajectories during the last 10 ns of a 100 ns MD simulations in the NTP ensemble and variant transfer integrals (*t*_1_ and *t*_2_) at the 100 ns of MD simulations. The calculated B‐factors show the temperature dependence of thermal atomic fluctuations between different molecules, which are expressed as $B=\frac{8}{3}\;\pi^{2}\Delta_{i}^{2}$, where Δ_*i*_ is the root mean square fluctuations (RMSF) of atom *i* (see Supporting Information for more detailed descriptions). b) Variant *t* value distributions and standard deviations (*σ*) revealing the magnitude of dynamic fluctuations.

The detrimental effect of molecular fluctuations of OSCs on charge transport has been studied in recent years,^[^ ^48^, ^49^, ^50^ ^]^ and the negatively affected charge transport due to fluctuations of the *π*‐electron cores is known as dynamic disorder.^[^ ^51^, ^52^ ^]^ Here, we investigate the interlayer interactions and molecular fluctuations of PhC_2_–BQQDI–C*_n_* using MD simulations, with constant number of molecules (N), temperature (T) and pressure (P) (isothermal‐isobaric, NTP ensemble). In the previous study, we have demonstrated small molecular fluctuations of PhC_2_–BQQDI (**Figure** **4**a) attributed to strong intralayer *π*‐electron core and interlayer phenyl‐to‐phenyl interactions. Surprisingly, despite having the molecularly flexible alkyl group, the interlayer chains of asymmetric PhC_2_–BQQDI–C_5_ also show similarly small degree of molecular fluctuations (small B‐factors) as PhC_2_–BQQDI (Figure 4a), which is possibly due to the stabilization effect by the “aromatic pocket” (Figure S11, Supporting Information), and the *π*‐electron core of PhC_2_–BQQDI–C_5_ also shows small degree of fluctuations. Based on this result, we argue that the alkyl chain PhC_2_–BQQDI–C_5_ does not behave as an ordinary flexible alkyl chain, but it rather mimics a structurally rigid phenyl group, which leads to small amplitude of molecular fluctuations. Similarly, PhC_2_–BQQDI–C_6_ also exhibits small amplitude of molecular fluctuations in the *π*‐electron cores, but the alkyl chains show noticeably large B‐factors and destabilization of the molecular mimicry conformation (Figure S16, Supporting Information). PhC_2_–BQQDI–C_7_ expectedly demonstrates large amplitude of molecular fluctuations in the alkyl chains, and the molecular mimicry in the single‐crystal structure is no longer retained in a large number of molecules during the MD simulations (Figure S16, Supporting Information). In addition, the *π*‐electron cores of PhC_2_–BQQDI–C_7_ show larger B‐factors that could potentially affect the charge‐transport capability.

Variant *t* values are calculated to understand the effect of dynamic disorder in the *π*–*π* stacking directions of PhC_2_–BQQDI–C*_n_* based on MD simulations. We extracted more than 500 dimers using their atomic coordinates at 100 ns acquired by the MD simulations to calculate their *t*_1‐3_ values. PhC_2_–BQQDI–C_5_ exhibits the smallest standard deviations (*σ*) of 23.0 and 13.2 meV in *t*_1_ and *t*_2_ directions, respectively (Figure 4b), suggesting that the charge‐transport capability is resilient to molecular fluctuations. On the other hand, PhC_2_–BQQDI–C*_n_* (*n* = 6 and 7) show larger *σ* of *t* values due to their molecular fluctuations (Figure S16, Supporting Information), indicating potentially compromised charge‐transport capabilities. In addition, it has been reported that the ratio of *σ* and averaged *t* values (*σ*/*t*_Avg._) quantifies the effect of dynamic orders.^[^ ^53^ ^]^ We take into consideration of averaged *t* and *σ* in all three charge‐transport directions (*t*_1_, *t*_2_, and *t*_3_) and obtain the ratio *σ*/*t*_(1,2,3)Avg._ to quantify the overall dynamic disorders. PhC_2_–BQQDI–C_5_ shows a *σ*/*t*_(1,2,3)Avg._ of 0.37, much smaller than 0.45 of PhC_2_–BQQDI–C_6_ and –C_7_, and slightly higher than 0.31 of the high‐performance PhC_2_–BQQDI (Figure 4b). Thus, PhC_2_–BQQDI–C_5_ may demonstrate resilience to dynamic disorders and promising OSC performances.

### 2.5 OFET Performances

**Figure 5:**
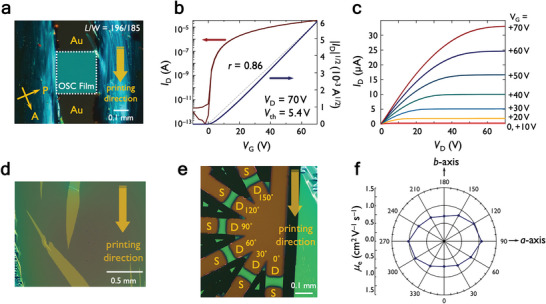
a) Polarized microscopic image of single‐crystalline thin‐film OFET. b) Transfer (black and gray dashed lines represent the fit to |*I*_D_|^1/2^ and the slope of an electrically ideal OFET,^[^ ^54^ ^]^ respectively) characteristics. c) Output characteristics evaluated under ambient conditions. d) Centimeter‐scale single‐crystalline thin film fabricated via the solution‐processed printing method; e) fan‐shaped OFETs on the centimeter‐scale single‐crystalline thin film with channels in every 30˚ relative to the printing direction (*L* = ∼40 µm, *W* = ∼90 µm); f) the resulting azimuthal *µ*.

To evaluate n‐type OSC performances of PhC_2_–BQQDI–C*_n_* (*n* = 5, 6, and 7) under ambient conditions, we fabricate bottom‐gate/top‐contact single‐crystalline thin‐film OFETs via the edge‐casting method.^[^ ^55^ ^]^ Solution‐processed temperatures for PhC_2_–BQQDI–C*_n_* ranged from 90–115 ˚C, which are lower than that of PhC_2_–BQQDI (130 ˚C),^[^ ^28^ ^]^ due to their higher solubilities (Table S3, Supporting Information). We confirm that the single crystalline thin films of PhC_2_–BQQDI–C*_n_* are consistent with their bulk single crystal structures to correlate their estimated charge‐transport capabilities and device performances (Figures S13–15, Supporting Information). PhC_2_–BQQDI–C_5_ demonstrates excellent single‐crystalline thin films (**Figure** **5**a) on polymeric gate dielectrics with apparent electron *µ* ranging between 1.1 and 1.9 cm^2^ V^−1^ s^−1^, where OFETs exhibiting *µ* greater than 1.5 cm^2^ V^−1^ s^−1^ are accompanied by relatively large threshold voltages (*V*_th_) and low reliability factor (*r*)^[^ ^54^ ^]^ typically less than 0.5 (Figure S19 and Table S4, Supporting Information). PhC_2_–BQQDI–C_5_ also shows an averaged *µ* of 1.59 cm^2^ V^−1^ s^−1^ (standard deviation: 0.22) over 45 devices using the same fabrication method (Figure S19, Supporting Information). The most textbook‐like OFET characteristics demonstrated an apparent *µ* of 1.2 cm^2^ V^−1^ s^−1^ and with an *r* of 0.86, which leads to an effective *µ* of 1.0 cm^2^ V^−1^ s^−1^ (Figure 5b,c). PhC_2_–BQQDI–C_6_ with the similar charge‐transport capability and exhibits an apparent *µ* of 1.2 cm^2^ V^−1^ s^−1^, though it displays a non‐ideal transfer curve with a low *r* of 0.28 and an effective *µ* of 0.33 cm^2^ V^−1^ s^−1^ (Figure S18a, Supporting Information). Similarly, PhC_2_–BQQDI–C_7_ demonstrates nonlinearity in its transfer characteristic, its highest apparent *µ* of 1.0 cm^2^ V^−1^ s^−1^ is accompanied by a low *r* of 0.35 and an effective *µ* of 0.35 cm^2^ V^−1^ s^−1^ (Figure S18b, Supporting Information). These OFET properties as well as the images of solution‐processed thin films (Figure S22, Supporting Information) imply a correlation between the disordering of alkyl chains and OFET performances. The device performances of current asymmetric and previous symmetric BQQDI derivatives are summarized in Table S4 (Supporting Information) for comparison. PhC_2_−BQQDI−C_5_ further shows good long‐term air stability (Figure S20, Supporting Information) and thermal‐stress durability (Figure S21, Supporting Information), comparable with the reported PhC_2_−BQQDI^[^ ^28^ ^]^ due to the low‐lying LUMO and structural stability.

Considering the excellent OFET performance of PhC_2_–BQQDI–C_5_, we successfully fabricated centimeter‐scale single‐crystalline thin films using the continuous edge‐casting method (Figure 5d; Figure S23, Supporting Information).^[^ ^56^, ^57^ ^]^ The excellent processability can be attributed to its adequate solubility and high crystallinity with negligible disordering of alkyl chains in the aggregated structure. The current single‐crystalline thin film size is two orders of magnitude larger than that of PhC_2_–BQQDI, and the *µ* of PhC_2_–BQQDI–C_5_ is similar to the former, suggesting its superior solution‐processability for applicable electronics. Herein, the large‐area single‐crystalline thin films of PhC_2_–BQQDI–C_5_ also allows us to study the directionality of its *µ* and correlate it with the estimated angle‐dependent inversed m*. This study has rarely been performed on n‐type OSCs due to their limited single‐crystalline sizes.^[^ ^58^ ^]^ We examined the device performance as well as anisotropic *µ* of the large‐area single‐crystalline thin film (Figure 5e). From the plotted azimuthal *µ* (Figure 5f; Figure S24, Supporting Information), an ellipsoid‐shape directionality of *µ* is apparent, where the highest *µ* is approximately along the *a*‐axis direction, which is inconsistent with the azimuthal inversed m* shown in Figure 3c. This might arise from the crystallographic feature. Owing to the *Pn* space group, the glide planes provide two different layer structures alternately stacked along the *c*‐axis direction, which afford mirrored characters of the azimuthal inversed m* (Figure S25a,b, Supporting Information). While neither of their shapes match the experimental anisotropic *µ*, interestingly, a similar shape is obtained when the inversed m* values are averaged at each angle (Figure S25c, Supporting Information). These results may imply a significant carrier distribution even in the second layer, although further studies are required for in‐depth understandings.

## 3 Conclusions

In summary, we have developed an effective and efficient synthetic strategy for asymmetric PhC_2_–BQQDI–C*_n_* (*n* = 5, 6 and 7) derivatives, showing high solubility compared to PhC_2_–BQQDI. The intriguing molecular mimicry of alkyl chains in the single‐crystal structure of PhC_2_–BQQDI–C_5_ with the *gauche* conformation has shown to be energetically favorable and persistent at elevated temperatures. The molecular mimicry geometry of PhC_2_–BQQDI–C_5_ results in small degree of molecular fluctuations. From the results of variant *t* calculations, PhC_2_–BQQDI–C_5_ shows the smallest *σ* of *t* values, indicating the resilience of the overall molecular assembly to dynamic disorders. We demonstrate that the large‐area single‐crystalline thin films enabled by PhC_2_–BQQDI–C_5_ allow us an in‐depth investigation of charge transport in n‐type OSCs, which is promising for developments of printed single‐crystalline organic electronics.

## Conflict of Interest

The authors declare no conflict of interest.

## Supporting information

Supporting Information

## Data Availability

The data that support the findings of this study are available in the supplementary material of this article.

## References

[1] V. Podzorov , E. Menard , A. Borissov , V. Kiryukhin , J. A. Rogers , M. E. Gershenson , Phys. Rev. Lett. 2004, 93, 086602. 15447211 10.1103/PhysRevLett.93.086602

[2] V. C. Sundar , J. Zaumseil , V. Podzorov , E. Menard , R. L. Willett , T. Someya , M. E. Gershenson , J. A. Rogers , Science 2004, 303, 1644. 15016993 10.1126/science.1094196

[3] A. L. Briseno , S. C. B. Mannsfeld , M. M. Ling , S. Liu , R. J. Tseng , C. Reese , M. E. Roberts , Y. Yang , F. Wudl , Z. Bao , Nature 2006, 444, 913. 17167482 10.1038/nature05427

[4] R. W. I. de Boer , T. M. Klapwijk , A. F. Morpurgo , Appl. Phys. Lett. 2003, 83, 4345.

[5] V. Podzorov , S. E. Sysoev , E. Loginova , V. M. Pudalov , M. E. Gershenson , Appl. Phys. Lett. 2003, 83, 3504.

[6] X. Yu , V. Kalihari , C. D. Frisbie , N. K. Oh , J. A. Rogers , Appl. Phys. Lett. 2007, 90, 162106.

[7] J. Takeya , J. Kato , K. Hara , M. Yamagishi , R. Hirahara , K. Yamada , Y. Nakazawa , S. Ikehata , K. Tsukagoshi , Y. Aoyagi , T. Takenobu , Y. Iwasa , Phys. Rev. Lett. 2007, 98, 196804. 17677647 10.1103/PhysRevLett.98.196804

[8] H. Minemawari , T. Yamada , H. Matsui , J. Y. Tsutsumi , S. Haas , R. Chiba , R. Kumai , T. Hasegawa , Nature 2011, 475, 364. 21753752 10.1038/nature10313

[9] Y. Diao , B. C. K. Tee , G. Giri , J. Xu , D. H. Kim , H. A. Becerril , R. M. Stoltenberg , T. H. Lee , G. Xue , S. C. B. Mannsfeld , Z. Bao , Nat. Mater. 2013, 12, 665. 23727951 10.1038/nmat3650

[10] A. Yamamura , S. Watanabe , M. Uno , M. Mitani , C. Mitsui , J. Tsurumi , N. Isahaya , Y. Kanaoka , T. Okamoto , J. Takeya , Sci. Adv. 2018, 4, eaao5758. 29423445 10.1126/sciadv.aao5758 PMC5804585

[11] T. Makita , S. Kumagai , A. Kumamoto , M. Mitani , J. Tsurumi , R. Hakamatani , M. Sasaki , T. Okamoto , Y. Ikuhara , S. Watanabe , J. Takeya , Proc. Natl. Acad. Sci. USA 2020, 117, 80. 31857386 10.1073/pnas.1909932116 PMC6955328

[12] H. Iino , T. Usui , J. Hanna , Nat. Commun. 2015, 6, 6828. 25857435 10.1038/ncomms7828 PMC4403349

[13] G. Kitahara , S. Inoue , T. Higashino , M. Ikawa , T. Hayashi , S. Matsuoka , S. Arai , T. Hasegawa , Sci. Adv. 2020, 6, abc8847. 10.1126/sciadv.abc8847 PMC7541062 33028533

[14] Z. He , D. S. H. S. Dai , M. Chen , D. Zou , G. K. K. Chik , R. Rafael , K. H. Lee , Y. Piao , S. Zhang , X. Cheng , P. K. L. Chan , Adv. Funct. Mater. 2022, 32, 2205129.

[15] T. Kubo , R. Häusermann , J. Tsurumi , J. Soeda , Y. Okada , Y. Yamashita , N. Akamatsu , A. Shishido , C. Mitsui , T. Okamoto , S. Yanagisawa , H. Matsui , J. Takeya , Nat. Commun. 2016, 7, 11156. 27040501 10.1038/ncomms11156 PMC4822010

[16] B. Crone , A. Dodabalapur , Y. Y. Lin , R. W. Filas , Z. Bao , A. LaDuca , R. Sarpeshkar , H. E. Katz , W. Li , Nature 2000, 403, 521. 10676955 10.1038/35000530

[17] H. Klauk , U. Zschieschang , J. Pflaum , M. Halik , Nature 2007, 445, 745. 17301788 10.1038/nature05533

[18] E. C. P. Smits , S. G. J. Mathijssen , P. A. van Hal , S. Setayesh , T. C. T. Geuns , K. A. H. A. Mutsaers , E. Cantatore , H. J. Wondergem , O. Werzer , R. Resel , M. Kemerink , S. Kirchmeyer , A. M. Muzafarov , S. A. Ponomarenko , B. de Boer , P. W. M. Blom , D. M. de Leeuw , Nature 2008, 455, 956.

[19] C. Wang , H. Dong , W. Hu , Y. Liu , D. Zhu , Chem. Rev. 2012, 112, 2208. 22111507 10.1021/cr100380z

[20] K. Takimiya , S. Shinamura , I. Osaka , E. Miyazaki , Adv. Mater. 2011, 23, 4347. 21842474 10.1002/adma.201102007

[21] T. Okamoto , C. P. Yu , C. Mitsui , M. Yamagishi , H. Ishii , J. Takeya , J. Am. Chem. Soc. 2020, 142, 9083. 32293879 10.1021/jacs.9b10450

[22] C. Mitsui , T. Okamoto , M. Yamagishi , J. Tsurumi , K. Yoshimoto , K. Nakahara , J. Soeda , Y. Hirose , H. Sato , A. Yamano , T. Uemura , J. Takeya , Adv. Mater. 2014, 26, 4546. 24811889 10.1002/adma.201400289

[23] N. Kasuya , J. Tsurumi , T. Okamoto , S. Watanabe , J. Takeya , Nat. Mater. 2021, 20, 1401. 34489565 10.1038/s41563-021-01074-4

[24] B. A. Jones , M. J. Ahrens , M. H. Yoon , A. Facchetti , T. J. Marks , M. R. Wasielewski , Angew. Chem., Int. Ed. 2004, 43, 6363. 10.1002/anie.200461324 15558692

[25] B. A. Jones , A. Facchetti , M. R. Wasielewski , T. J. Marks , J. Am. Chem. Soc. 2007, 129, 15259. 17999505 10.1021/ja075242e

[26] C. P. Yu , R. Kimura , T. Kurosawa , E. Fukuzaki , T. Watanabe , H. Ishii , S. Kumagai , M. Yano , J. Takeya , T. Okamoto , Org. Lett. 2019, 21, 4448. 31184164 10.1021/acs.orglett.9b01239

[27] X. Chen , Y. He , M. U. Ali , Y. He , Y. Zhu , A. Li , C. Zhao , I. F. Perepichka , H. Meng , Sci. China: Chem. 2019, 62, 1360.

[28] T. Okamoto , S. Kumagai , E. Fukuzaki , H. Ishii , G. Watanabe , N. Niitsu , T. Annaka , M. Yamagishi , Y. Tani , H. Sugiura , T. Watanabe , S. Watanabe , J. Takeya , Sci. Adv. 2020, 6, eaaz0632. 32494668 10.1126/sciadv.aaz0632 PMC7195148

[29] S. Kumagai , S. Watanabe , H. Ishii , N. Isahaya , A. Yamamura , T. Wakimoto , H. Sato , A. Yamano , T. Okamoto , J. Takeya , Adv. Mater. 2020, 32, 2003245. 10.1002/adma.202003245 33191541

[30] C. P. Yu , N. Kojima , S. Kumagai , T. Kurosawa , H. Ishii , G. Watanabe , J. Takeya , T. Okamoto , Commun Chem 2021, 4, 155. 36697635 10.1038/s42004-021-00583-2 PMC9814529

[31] S. Kumagai , H. Ishii , G. Watanabe , C. P. Yu , S. Watanabe , J. Takeya , T. Okamoto , Acc. Chem. Res. 2022, 55, 660. 35157436 10.1021/acs.accounts.1c00548

[32] J. Rivnay , L. H. Jimison , J. E. Northrup , M. F. Toney , R. Noriega , S. Lu , T. J. Marks , A. Facchetti , A. Salleo , Nat. Mater. 2009, 8, 952. 19898460 10.1038/nmat2570

[33] A. S. Molinari , H. Alves , Z. Chen , A. Facchetti , A. F. Morpurgo , J. Am. Chem. Soc. 2009, 131, 2462. 19191497 10.1021/ja809848y

[34] M. Gsänger , J. H. Oh , M. Könemann , H. W. Höffken , A. M. Krause , Z. Bao , F. Würthner , Angew. Chem., Int. Ed. 2010, 49, 740. 10.1002/anie.200904215 19882605

[35] A. Nowak‐Król , K. Shoyama , M. Stolte , F. Würthner , Chem. Commun. 2018, 54, 13763. 10.1039/c8cc07640e 30465555

[36] W. Pisula , M. Kastler , D. Wasserfallen , T. Pakula , K. Müllen , J. Am. Chem. Soc. 2004, 126, 8074. 15225022 10.1021/ja048351r

[37] H. Minemawari , M. Tanaka , S. Tsuzuki , S. Inoue , T. Yamada , R. Kumai , Y. Shimoi , T. Hasegawa , Chem. Mater. 2017, 29, 1245.

[38] S. Arai , S. Inoue , T. Hamai , R. Kumai , T. Hasegawa , S. Arai , T. Hamai , T. Hasegawa , S. Inoue , R. Kumai , Adv. Mater. 2018, 30, 1707256. 10.1002/adma.201707256 29691910

[39] Y. Che , A. Datar , K. Balakrishnan , L. Zang , J. Am. Chem. Soc. 2007, 129, 7234. 17506565 10.1021/ja071903w

[40] C. Xue , R. Sun , R. Annab , D. Abadi , S. Jin , Tetrahedron Lett. 2009, 50, 853.

[41] R. K. Gupta , A. S. Achalkumar , J. Org. Chem. 2018, 83, 6290. 29772902 10.1021/acs.joc.8b00161

[42] C. S. Sample , E. Goto , N. v Handa , Z. A. Page , Y. Luo , C. J. Hawker , J Mater Chem C Mater 2017, 5, 1052.

[43] L. Trembleau , J. Rebek , Science 2003, 301, 1219. 12947192 10.1126/science.1086644

[44] M. Mravic , J. L. Thomaston , M. Tucker , P. E. Solomon , L. Liu , W. F. DeGrado , Science 2019, 363, 1418. 30923216 10.1126/science.aav7541 PMC7380683

[45] K. Kato , H. Tanaka , Adv Phys X 2016, 1, 55.

[46] K. Kato , Y. Tanaka , M. Yamauchi , K. Ohara , T. Hatsui , J Synchrotron Radiat 2019, 26, 762. 31074441 10.1107/S1600577519002145 PMC6510202

[47] S. Inoue , H. Minemawari , J. Tsutsumi , M. Chikamatsu , T. Yamada , S. Horiuchi , M. Tanaka , R. Kumai , M. Yoneya , T. Hasegawa , Chem. Mater. 2015, 27, 3809.

[48] A. S. Eggeman , S. Illig , A. Troisi , H. Sirringhaus , P. A. Midgley , Nat. Mater. 2013, 12, 1045. 23892786 10.1038/nmat3710

[49] S. Illig , A. S. Eggeman , A. Troisi , L. Jiang , C. Warwick , M. Nikolka , G. Schweicher , S. G. Yeates , Y. Henri Geerts , J. E. Anthony , H. Sirringhaus , Nat. Commun. 2016, 7, 10736. 26898754 10.1038/ncomms10736 PMC4764867

[50] G. Schweicher , G. D'Avino , M. T. Ruggiero , D. J. Harkin , K. Broch , D. Venkateshvaran , G. Liu , A. Richard , C. Ruzié , J. Armstrong , A. R. Kennedy , K. Shankland , K. Takimiya , Y. H. Geerts , J. A. Zeitler , S. Fratini , H. Sirringhaus , Adv. Mater. 2019, 31, 1902407. 10.1002/adma.201902407 31512304

[51] A. Troisi , J. Chem. Phys. 2011, 134, 034702. 21261379 10.1063/1.3524314

[52] A. Fratini , S. Ciuchi , D. Mayou , G. T. de Laissardière , A. Troisi , Nat. Mater. 2017, 16, 998. 28892051 10.1038/nmat4970

[53] T. Fukami , H. Ishii , N. Kobayashi , T. Uemura , K. Sakai , Y. Okada , J. Takeya , K. Hirose , Appl. Phys. Lett. 2015, 106, 143302.

[54] H. H. Choi , K. Cho , C. D. Frisbie , H. Sirringhaus , V. Podzorov , Nat. Mater. 2017, 17, 2. 29255225 10.1038/nmat5035

[55] J. Takeya , M. Yamagishi , Y. Tominari , R. Hirahara , Y. Nakazawa , T. Nishikawa , T. Kawase , T. Shimoda , S. Ogawa , Appl. Phys. Lett. 2007, 90, 102120.

[56] J. Soeda , T. Uemura , T. Okamoto , C. Mitsui , M. Yamagishi , J. Takeya , Appl. Phys. Express 2013, 6, 076503.

[57] S. Kumagai , A. Yamamura , T. Makita , J. Tsurumi , Y. Y. Lim , T. Wakimoto , N. Isahaya , H. Nozawa , K. Sato , M. Mitani , T. Okamoto , S. Watanabe , J. Takeya , Sci. Rep. 2019, 9, 15897. 31685835 10.1038/s41598-019-50294-x PMC6828694

[58] T. He , Y. Wu , G. D'Avino , E. Schmidt , M. Stolte , J. Cornil , D. Beljonne , P. P. Ruden , F. Würthner , C. D. Frisbie , Nat. Commun. 2018, 9, 2141. 29849022 10.1038/s41467-018-04479-z PMC5976653

