# Supplementary material for: Asymmetrically Functionalized Electron‐Deficient π‐Conjugated System for Printed Single‐Crystalline Organic Electronics
Source: Adv Sci (Weinh). 2023 Sep 15;10(29):2207440. doi: 10.1002/advs.202207440 (PMC10582418; doi:10.1002/advs.202207440)
Supplement: Supplementary file 1 — Supporting Information [file ADVS-10-2207440-s001.pdf]

ADVANCED SCIENCE
Open Access

# Supporting Information

for *Adv. Sci.*, DOI 10.1002/advs.202207440

Asymmetrically Functionalized Electron-Deficient $\pi$-Conjugated System for Printed Single-Crystalline Organic Electronics

*Craig P. Yu, Shohei Kumagai, Michitsuna Tsutsumi, Tadanori Kurosawa, Hiroyuki Ishii, Go Watanabe, Daisuke Hashizume, Hiroki Sugiura, Yukio Tani, Toshihiro Ise, Tetsuya Watanabe, Hiroyasu Sato, Jun Takeya and Toshihiro Okamoto**

# Supporting Information

## **Asymmetrically Functionalized Nitrogen-Containing $\pi$-Conjugated System for n-Type Printed Single-Crystalline Organic Electronics**

Craig P. Yu,[1] Shohei Kumagai,[1] Michitsuna Tsutsumi,[1] Tadanori Kurosawa,[1] Hiroyuki Ishii,[2] Go Watanabe,[3] Daisuke Hashizume,[4] Hiroki Sugiura,[5] Yukio Tani,[5] Toshihiro Ise,[5] Tetsuya Watanabe,[5] Hiroyasu Sato,[6] Jun Takeya,[1,7] Toshihiro Okamoto[1,8]*

[1]Material Innovation Research Center (MIRC) and Department of Advanced Materials Science, Graduate School of Frontier Sciences, The University of Tokyo, 5-1-5 Kashiwanoha, Kashiwa, Chiba 277-8561, Japan

[2]Department of Applied Physics, Faculty of Pure and Applied Sciences, University of Tsukuba, 1-1-1 Tennodai, Tsukuba, Ibaraki 305-8573, Japan

[3]Department of Physics, School of Science, Kitasato University, 1-15-1 Kitasato, Minami-ku, Sagamihara, Kanagawa 252-0373, Japan.

[4]RIKEN Center for Emergent Matter Science (CEMS), 2-1 Hirosawa, Wako, Saitama 351-0198, Japan

[5]FUJIFILM Corp., 577 Ushijima, Kaisei-machi, Ashigarakami-gun, Kanagawa 258-8577, Japan

[6]Rigaku Corp., 3-9-12 Matsubara-cho, Akishima, Tokyo 196-8666, Japan

[7]International Center for Materials Nanoarchitectonics (MANA), National Institute for Materials Science (NIMS), 1-1 Namiki, Tsukuba 205-0044, Japan

[8]PRESTO, JST, 4-1-8 Honcho, Kawaguchi, Saitama 332-0012, Japan

*Corresponding Author:

Toshihiro Okamoto, tokamoto@k.u-tokyo.ac.jp

## Table of Contents


1. **Materials and General Characterization Methods** ........ 21

2. **Synthetic Procedures** ........ 22

3. **Theoretical Calculations** ........ 24

4. **$^1$H and $^{13}$C NMR Spectra** ........ 25

5. **Fundamental Properties** ........ 30

   *Thermal Properties* ........ 30

   *Electrochemical Measurements* ........ 31

   *Solution-State UV-vis Absorbance* ........ 32

   *Optical Gap Estimated from the Tauc Plots* ........ 33

6. **X-Ray Crystallography** ........ 34

   *Alkyl Chain Conformation* ........ 36

   *Temperature-Variant Powder X-Ray Diffractions (PXRD)* ........ 37

   *X-Ray Diffraction of Edge-Casted Thin-Films* ........ 38

7. **Molecular Dynamic Simulations** ........ 40

8. **Solubility** ........ 42

9. **OFET Fabrications** ........ 43

   *Preparation of OFET Substrates* ........ 43

   *Fabrications of Solution-Processed Single-Crystalline Thin Films* ........ 43

   *Fabrication of Large-Area Single-Crystalline Thin Films* ........ 43

   *OFET Measurements* ........ 44

10. **OFET Performance Evaluations** ........ 45

11. **Single-Crystalline Thin Films** ........ 51

12. **Large-Area Single-Crystalline Thin Film** ........ 52

13. **Effective Masses of Different Molecular Layers** ........ 53

14. **References** ........ 54


## 1. Materials and General Characterization Methods

Reagents and anhydrous solvents were purchased from Tokyo Chemical Industry Co., Ltd and Kanto Chemicals, respectively, and *o*–dichlorobenzene was purified by the solvent purification system prior to use. All reactions were carried out under an atmosphere of argon.

Analytical thin-layer chromatography (TLC) was performed on glass plates with silica gel containing fluorescent indicator (Merck TLC Silica gel 60 F254, 1 mm). Column chromatography was performed on Kanto silica gel 60. All HPLC spectra were recorded on a column by Phenomenex (Kinetex 5u C18 100A, New column 150×4.6 mm) with the mixture of solvent of THF: $H_2O$ = 6.2: 3.8, and the flow rate was 1.0 mL $min^{-1}$. UV-vis spectra were measured with a JASCO V-670 spectrometer. Diffuse reflectance spectra were acquired by diluting crystalline BQQDI samples with $BaSO_4$. $^{13}C$ NMR of compound **3** was recorded on an AVANCE 700 III, and all other NMR spectra were recorded on JEOL ECS400 spectrometer. Chemical shifts were reported in parts per million (ppm, d scale) from residual protons in the deuterated solvent for $^1H$ NMR (5.93 ppm for 1,1,2,2-tetrachloroethane-$d_2$ (TCE-$d_2$) and 7.26 ppm for chloroform-*d* ($CDCl_3$)) and $^{13}C$ NMR (73.78 ppm for 1,1,2,2-tetrachloroethane-$d_2$ (TCE-$d_2$) and 77.16 ppm for chloroform-*d* ($CDCl_3$)). The data were presented in the following format: chemical shift, multiplicity (s = singlet, d = doublet, t = triplet, quint = quintet, m = multiplet, br = broad, brm = broad multiplet), coupling constant in Hertz (Hz), signal area integration in natural numbers.

## 2. Synthetic Procedures

**Synthetic procedure for *N*-(4-methoxybenzyl)-phenethylamine**.

A flame-dried round-bottom flask was charged with anhydrous $CH_2Cl_2$ (208 mL), $MgSO_4$ (250 g, 2.08 mol, 10 equiv.), phenethylamine (25.3 g, 208 mmol, 1.0 equiv.) and *p*-anisaldehyde (28.4 g, 208 mmol, 1.0 equiv.). The mixture was stirred at room temperature for 2 h and filtered via vacuum filtration and the solvent was removed *in vacuo*. (*E*)-1-(4-methoxyphenyl)-*N*-phenethylmethanimine was obtained as a yellow liquid (43.3 g) and used without any purification. Subsequently, the obtained imine (43.3 g, 181 mmol, 1.0 equiv.), $NaBH_4$ (18.9 g, 500 mmol, 2.8 equiv.), anhydrous $CH_2Cl_2$ (362 mL), MeOH (362.48 mL) were added to a round-bottom flask under argon. The mixture was stirred at 0 °C for 2 h and the reaction was quenched by water. The compound was extracted with $CH_2Cl_2$ and 2M HCl (100 mL) was added to the organic layer and the precipitates were collected via filtration. The filtrates were dissolved in water (150 mL) and sodium carbonate was added until the white solids disappeared, and the compound was then extracted with $CH_2Cl_2$ (50 mL × 3). After $CH_2Cl_2$ was removed *in vacuo*, the title compound was obtained as a light-yellow liquid without further purification (36.1 g, 72% yield). $^1H$ NMR (400 MHz, $CDCl_3$): δ 7.31-7.19 (m, 7H), 6.87-6.83 (m, 2H), 3.77 (d, *J* = 17.6 Hz, 5H), 2.92-2.81 (m, 4H). The spectrum is in good agreement with the reported data[59].

**Synthetic procedure for 3,9-dimethyl 4-(2,4,6-trichlorophenyl) 10-((4-methoxybenzyl)(phenethyl)carbamoyl)benzo[*de*]isoquinolino[1,8-*gh*]quinoline-3,4,9-tricarboxylate (2)**.

A flame-dried three-neck round bottom flask equipped with an Ar inlet and reflex condenser was charged with 3,9-dimethyl 10,4-bis(2,4,6-trichlorophenyl)benzo[*de*]isoquinolino[1,8-*gh*]quinoline-3,4,9,10-tetracarboxylate (**1**) (2.45 g, 3.00 mmol, 1.0 equiv.) and anhydrous *o*-DCB (90 mL). After the complete dissolution of **1** at 180 °C, a solution of PMB amine (2.89 g, 12.0 mmol, 4.0 equiv.) was added in one portion to the system. The reaction was carried out at 180 °C and monitored by HPLC. After the peak area of **2** reached the maximum value (ca. 40 min), the mixture was cooled down to room temperature and the solvent was removed *in vacuo*. The crude product was purified by silica gel column chromatography using PhMe/EtOAc as eluents to afford compound **2** as an orange solid. (780 mg, 45% yield). $^1$H NMR (400 MHz, TCE-$d_2$, 100 ˚C): δ 9.23-8.84 (m, 5H), 8.73-7.65 (brm, 1H), 7.46(s, 2H), 7.43-6.82 (brm, 9H), 5.55-4.08 (brm, 2H), 4.08-3.93 (brm, 3H), 3.89 (s, 3H,), 3.82 (s, 3H), 3.75-3.62 (brm, 2H), 3.08-2.87 (brm, 2H). HRMS (APCl$^+$-TOF): Calcd for $C_{46}H_{32}Cl_3N_3O_8$ [M+H] 860.1333. Found, 860.1325. Elemental Analysis. Calcd for $C_{46}H_{32}Cl_3N_3O_8$: C 64.16; H 3.75; N 4.88. Found: C 64.02; H 3.87; N 4.90.

**General synthetic procedure for one-pot synthesis of PhC$_2$–BQQDI–C$_n$**.

A flame-dried Schlenk tube equipped with a stir bar was charged with compound **2** (400 mg, 1.0 equiv.), alkylamine (1.5 equiv.), and anhydrous *o*-DCB (0.05 M). The mixture was heated at 150 ˚C for one hour under argon and subsequently cooled to room temperature. To the dark red solution was added TfOH (2.5 equiv.) and the mixture was then stirred at 150 ˚C for three hours under argon. As the reaction completion was indicated by $^1$H NMR, the mixture was added dropwise to a stirring MeOH solution and the dark precipitates were collected via vacuum filtration. The products were recrystallized from *o*-DCB to afford the target compounds.

**Characterization of PhC$_2$–BQQDI–C$_5$**. 240 mg, 91% yield. $^1$H NMR (400 MHz, TCE-$d_2$, 100 ˚C): δ 9.65 (s, 2H), 9.28 (d, $J$ = 7.6 Hz, 2H), 8.84 (d, $J$ = 8.0 Hz, 2H), 7.34-7.19 (m, 5H), 4.45 (t, $J$ = 7.6 Hz, 8.0 Hz, 2H), 4.20 (t, $J$ = 7.2 Hz, 7.2 Hz, 2H), 3.08 (t, $J$ = 8.0 Hz, 7.2 Hz, 2H), 1.81 (quint, $J$ = 8.8 Hz, 6.0 Hz, 6.8 Hz, 6.0 Hz, 2H), 1.44-1.27 (m, 10H), 0.95 (t, $J$ = 6.4 Hz, 3H). HRMS (APCI$^+$-TOF): Calcd for $C_{35}H_{26}N_4O_4$ [M+H] 567.1954. Found 567.1958. Elemental Analysis. Calcd for $C_{35}H_{26}N_4O_4$: C 74.19; H 4.63; N 9.89. Found 74.17; H 4.73; N 9.80.

**Characterization of $PhC_2$–BQQDI–$C_6$.** 243 mg, 90% yield. $^1H$ NMR (400 MHz, TCE-$d_2$, 100 ˚C): δ 9.64 (s, 2H), 9.28 (d, $J$ = 7.6 Hz, 2H), 8.84 (d, $J$ = 8.0 Hz, 2H), 7.34-7.19 (m, 5H), 4.45 (t, $J$ = 7.2 Hz, 8.4 Hz, 2H), 4.20 (t, $J$ = 7.6 Hz, 7.2 Hz, 2H), 3.08 (t, $J$ = 7.2 Hz, 84 Hz), 1.78 (quint, $J$ = 8.0 Hz, 6.4 Hz, 7.2 Hz, 8.0 Hz, 2H), 1.47-1.27 (m, 6H), 0.92 (t, $J$ = 6.4 Hz, 6.8 Hz, 3H). HRMS ($APCI^+$-TOF): Calcd for $C_{36}H_{28}N_4O_4$ [M+H] 581.2111. Found 581.2124. Elemental Analysis. Calcd for $C_{36}H_{28}N_4O_4$: C 74.47; H 4.86; N 9.65. Found C 74.32; H 4.96; N 9.44.

**Characterization of $PhC_2$–BQQDI–$C_7$.** 254 mg, 92% yield. $^1H$ NMR (400 MHz, TCE-$d_2$, 100 ˚C): δ 9.64 (s, 2H), 9.27 (d, $J$ = 8.0 Hz, 2H), 8.84 (d, $J$ = 7.6 Hz, 2H), 7.34-7.19 (m, 5H), 4.45 (t, $J$ = 7.6 Hz, 8.4 Hz, 2H), 4.20 (t, $J$ = 7.2 Hz, 7.6 Hz, 2H), 3.08 (t, $J$ = 7.6 Hz, 8 Hz, 2H), 1.79 (quint, $J$ = 7.6 Hz, 7.2 Hz, 7.2 Hz, 7.6 Hz, 2H), 1.43-1.32 (m, 9H), 0.90 (t, $J$ = 6.8 Hz, 6.8 Hz, 3H). HRMS ($APCI^+$-TOF): Calcd for $C_{37}H_{30}N_4O_4$ [M+H] 595.2345. Found 595.2348. Elemental Analysis. Calcd for $C_{37}H_{30}N_4O_4$: C 74.73; H 5.09; N 9.42. Found C 74.71; H 5.16; N 9.22.

## 3. Theoretical Calculations

Theoretical calculations of transfer integral and effective mass were conducted using the GAMESS package[60]. The Kohn–Sham eigenstates of all compounds in this work were calculated at the PBEPBE/6-31G(d) level of theory. Transfer integrals ($t$) between LUMOs of neighboring molecules in the crystal structures were estimated by the dimer method[61]. To further understand the carrier transporting capabilities in the single-crystal state, their LUMO band structures $E(k)$ were calculated by the tight-binding approximation using transfer integrals. Intermolecular interaction energy between two adjacent molecules were obtained at the M06-2X/6-31++G(d,p) level of DFT with counterpoise correction for the basis set superposition error[62]. The calculations were performed using the Gaussian 09 program package[63].

## 4. $^1H$ and $^{13}C$ NMR Spectra

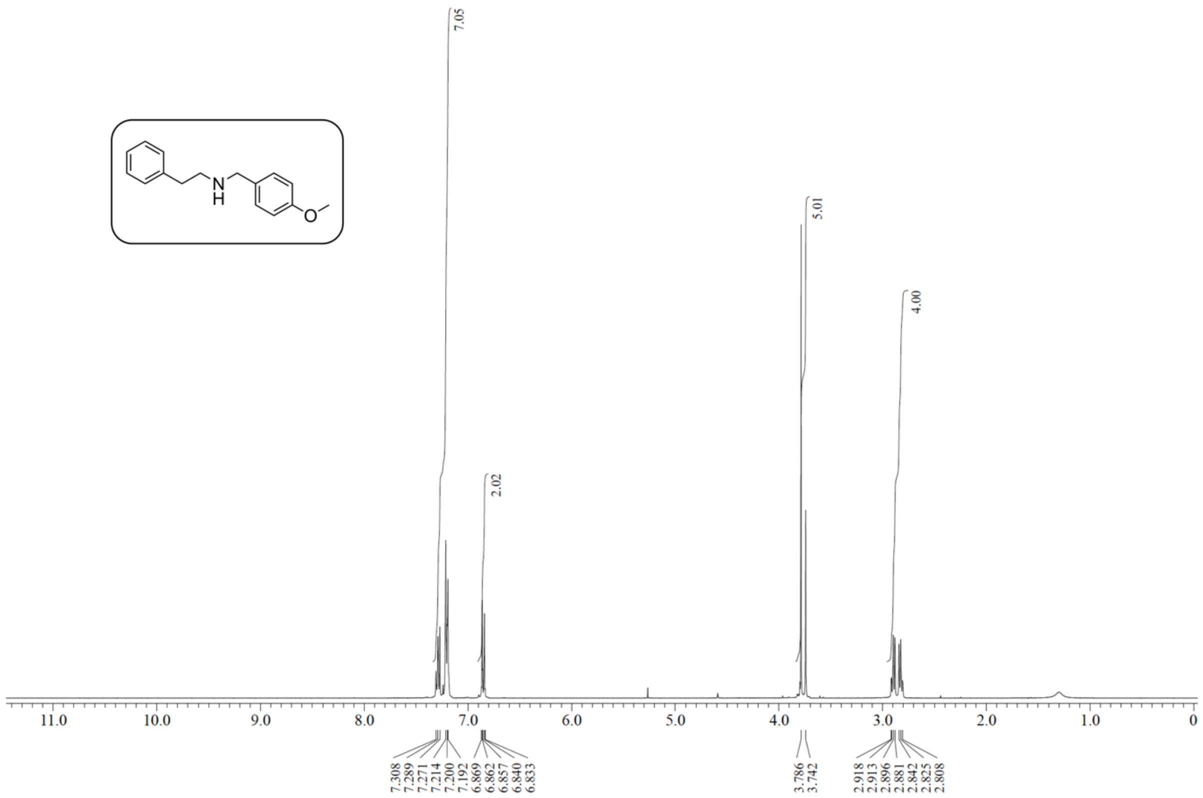

Figure S1. $^{1}$H NMR of *N*-(4-methoxybenzyl)-2-phenylethan-1-amine in $CDCl_3$.

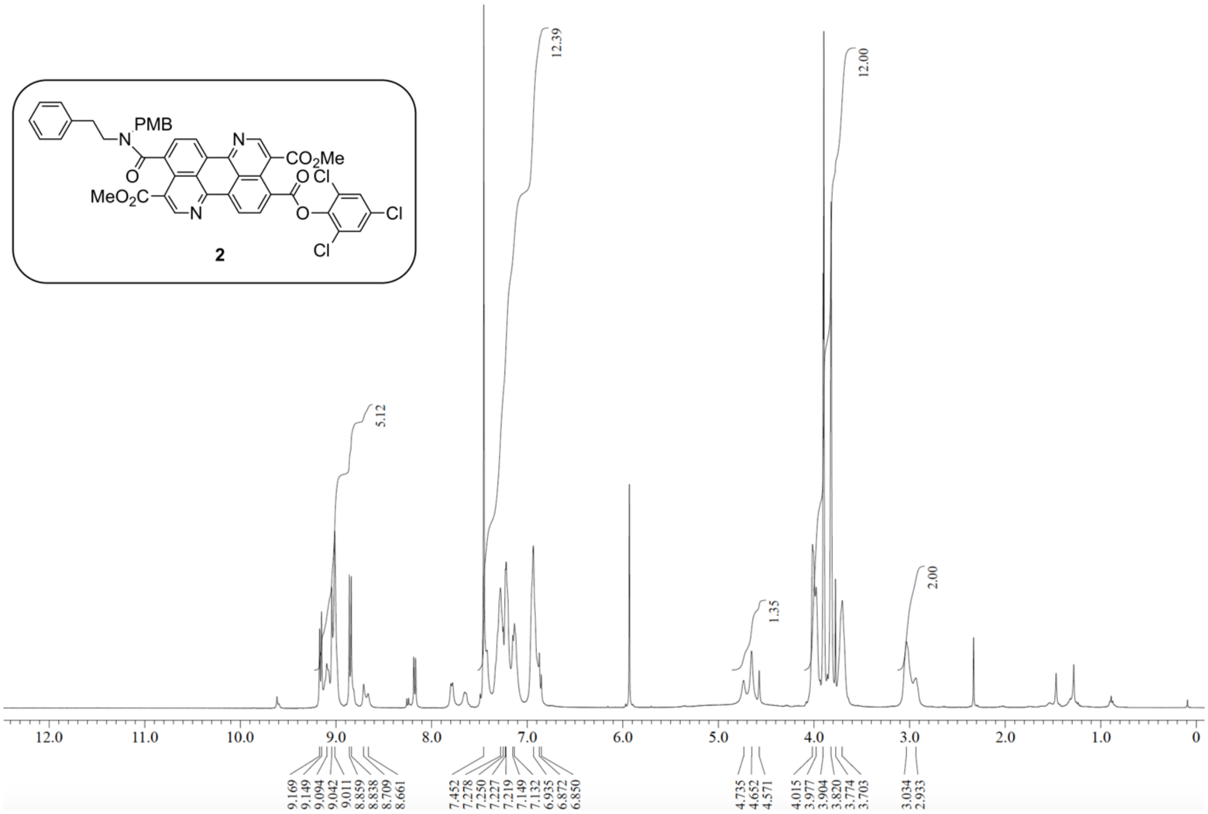

Figure S2. $^1$H NMR of compound **2** in TCE-$d_2$ at 100 ˚C.

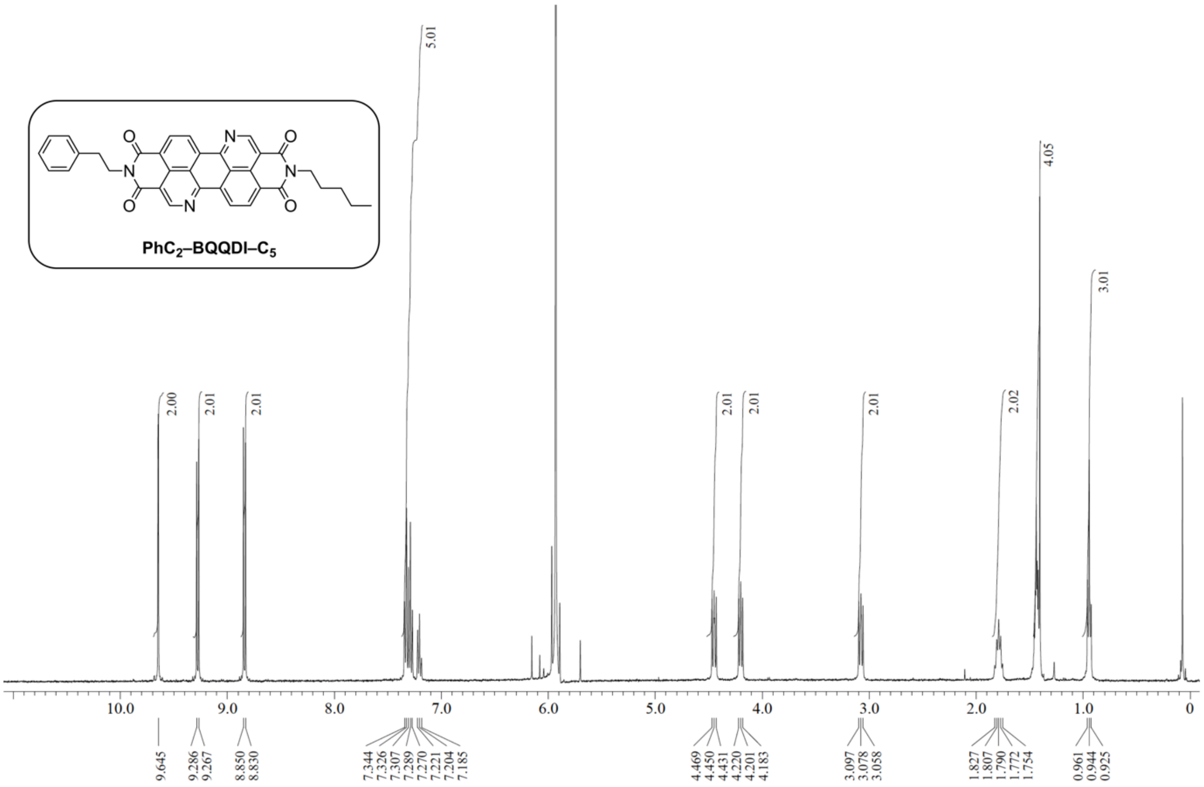


Figure S3. $^1$H NMR of compound PhC$_2$–BQQDI–C$_5$ in TCE-$d_2$ at 100 ˚C.

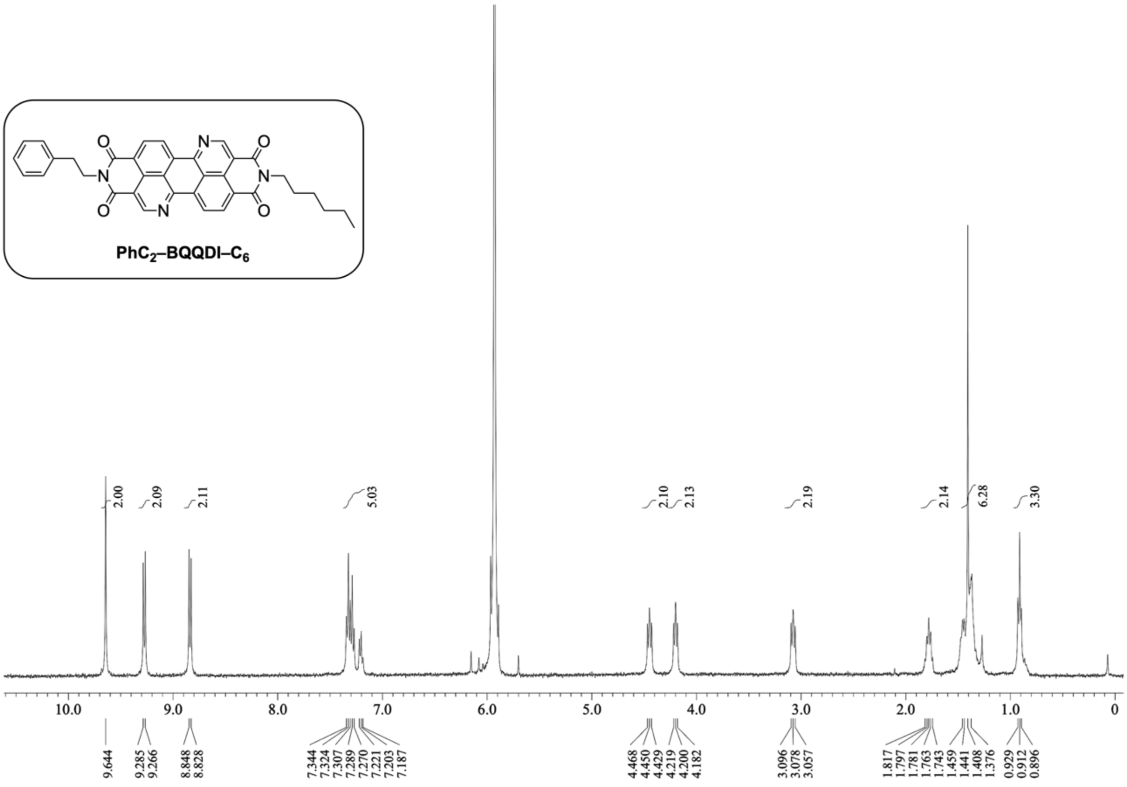

Figure S4. $^1$H NMR of compound $PhC_2$–BQQDI–$C_6$ in TCE-$d_2$ at 100 ˚C.

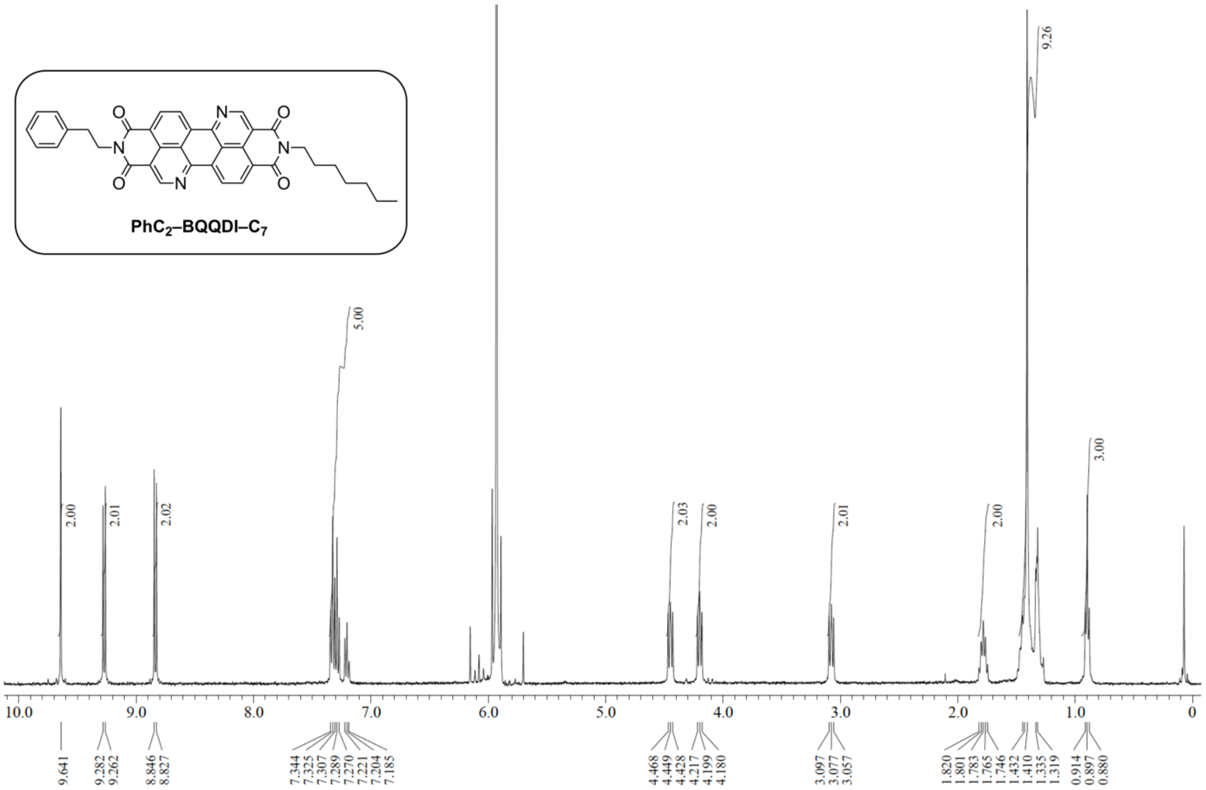


Figure S5. $^1$H NMR of compound $PhC_2$–BQQDI–$C_7$ in TCE-$d_2$ at 100 ˚C. Fundamental Properties

Thermogravimetric−differential thermal analysis was performed on a Rigaku Thermo Plus EVO II TG 8121 at a heating rate of 1 K $min^{-1}$ under a nitrogen flow of 100 mL $min^{-1}$. The differential scanning calorimetry measurements were performed with a Rigaku Thermo Plus EVO II DSC 8270 at a heating rate of 5 K $min^{-1}$ under a nitrogen flow of 50 mL $min^{-1}$.

Thermal Properties

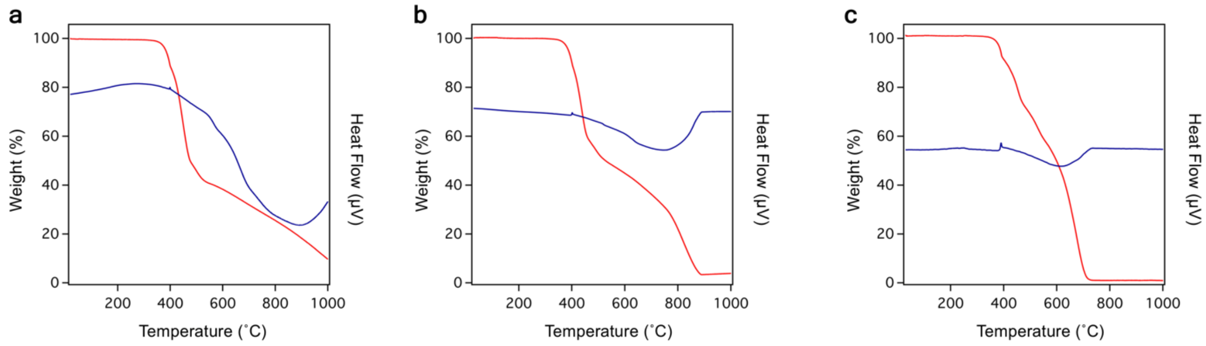


Figure S6. TG-DTA plots of **a** $PhC_2$–BQQDI–$C_5$, **b** $PhC_2$–BQQDI–$C_6$, and **c** $PhC_2$–BQQDI–$C_7$.

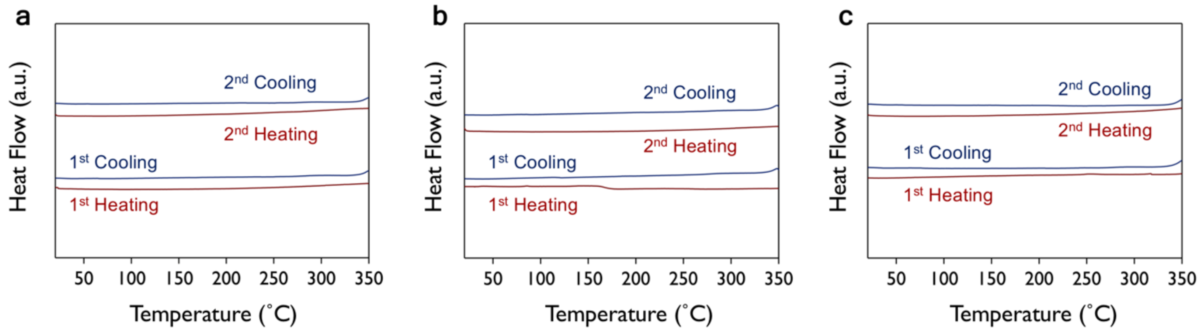


Figure S7. DSC plots of **a** $PhC_2$–BQQDI–$C_5$, **b** $PhC_2$–BQQDI–$C_6$, and **c** $PhC_2$–BQQDI–$C_7$.

Electrochemical Measurements

Cyclic voltammetry was conducted on a BAS electrochemical analyzer ALS 622D using a three-electrode cell with a glassy carbon as the working electrode, a Pt wire as the counter electrode and 0.01 M Ag/$AgNO_3$ (in benzonitrile containing 0.1 M tetrabutylammonium hexafluorophosphate ($TBAPF_6$)) as the reference electrode. The measurements were carried out under an argon atmosphere using a benzonitrile solution at 100 ˚C with a concentration of 0.25 mM, and 0.1 M $TBAPF_6$ as a supporting electrolyte with a scan rate of 0.1 V $s^{-1}$. The redox potentials were calibrated with ferrocene (Fc; $E(Fc/Fc^+) = 0$ V) as an internal standard. Benzonitrile was passed through a pad of aluminum oxide 60 for purification prior to use.

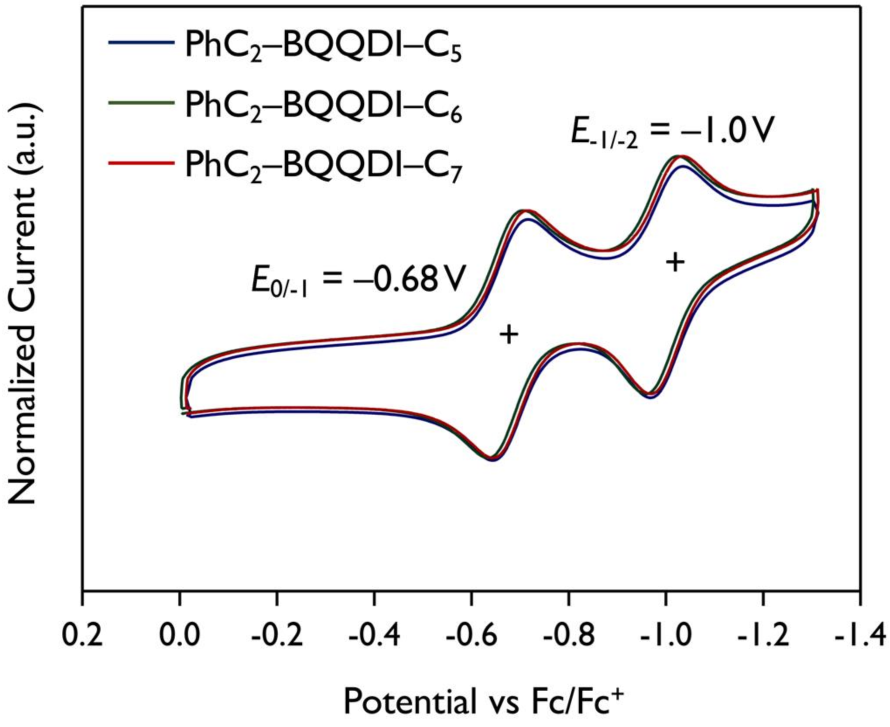

Figure S8. Cyclic voltammograms of PhC$_2$–BQQDI–C$_n$, where $E_{0/-1}$ and $E_{-1/-2}$ attribute to the half-wave reduction potentials corresponding to the first and second redox reactions, respectively (vs. Fc/Fc$^+$). $E_{LUMO}$ (vs. vacuum level) is estimated from $E_{0/-1}$: $E_{LUMO} = -4.8 - E_{0/-1}$.

Solution-State UV-vis Absorbance

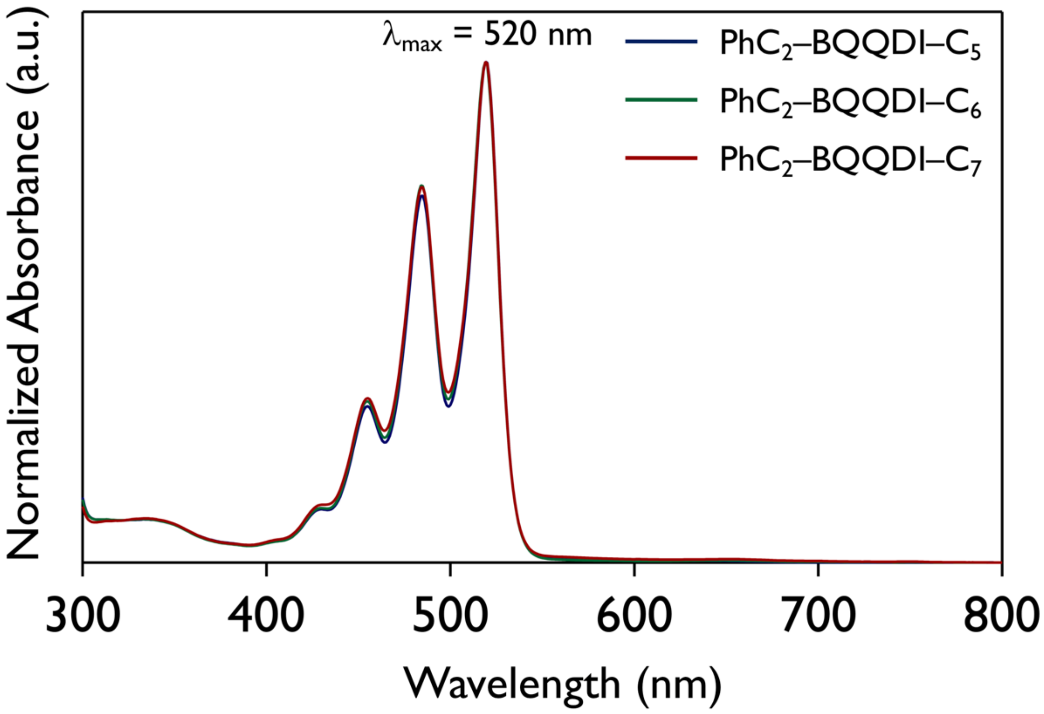


Figure S9. Normalized solution-state UV-vis absorbance of PhC$_2$–BQQDI–C$_n$ measured in benzonitrile at room temperature. Optical bandgap was estimated from the absorption edge at 545 nm.

Optical Gap Estimated from the Tauc Plots

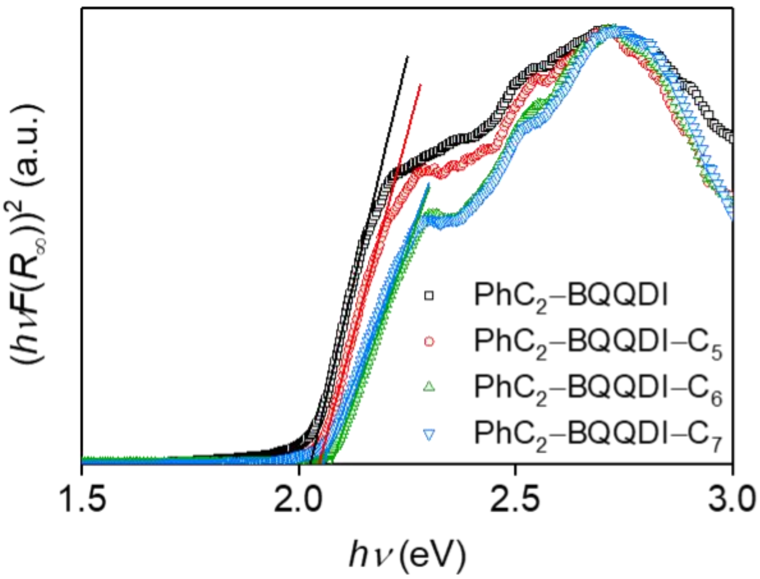


Figure S10. Tauc plot. $h\nu$: the photon energy; $F(R_\infty)$: Kubelka-Munk function. $(h\nu F(R_\infty))^2$ is normalized. Solid lines show the linear fitting.

Table S1. Optical gap estimated from the Tauc plots.

| Material | $PhC_2$–BQQDI | $PhC_2$–BQQDI–$C_5$ | $PhC_2$–BQQDI–$C_6$ | $PhC_2$–BQQDI–$C_7$ |
|---|---|---|---|---|
| $E_{opt}$ (eV) | 2.03 | 2.05 | 2.07 | 2.06 |

## 5. X-Ray Crystallography

$PhC_2$–BQQDI–$C_n$ single crystals were obtained by means of slow-cooling over a period of 48 hours in a mixture of nitrobenzene and 1–methylnaphthalene, and $PhC_2$–BQQDI–$C_5$ crystals were grown in the mixture of nitrobenzene and 1-chloronaphthalene. Single-crystal X-ray diffraction data were collected on either a Rigaku R-AXIS RAPID II imaging plate diffractometer with CuKα radiation (λ = 1.54187 Å) or a Rigaku XtaLAB Synergy-Custom instrument with CuKα radiation (λ = 1.54184 Å) at room temperature. The structures were solved by direct methods [SHELXT (2015)] and refined by full-matrix least-squares procedures on $F^2$ for all reflections [SHELXL (Ver. 2014/7) or SHELXL (Ver. 2018/3)]. While positions of all hydrogen atoms were calculated geometrically, and refined by applying riding model, all other atoms were refined anisotropically. Crystallographic data have been

deposited in the Cambridge Crystallographic Data Centre as a supplementary publication. These data can be obtained free of charge at www.ccdc.cam.ac.uk/data_request/cif. The temperature-variant PXRD studies of $PhC_2$–BQQDI–$C_5$ was carried out using the synchrotron X-ray powder diffraction with the wavelength of 0.8 Å at BL44B2 at SPring-8 RIKEN Materials Science Beamline (BL44B2)[45,46]. Powders of $PhC_2$–BQQDI–$C_5$ were packed in glass capillaries and the diffraction patterns are recorded in the temperature ranged from 25 to 200 ˚C. The intensities of diffraction patterns at different temperatures were normalized for better comparison.

Table S2. Single-crystal data of $PhC_2$–BQQDI–$C_n$.

| | **$PhC_2$–BQQDI–$C_5$** | **$PhC_2$–BQQDI–$C_6$** | **$PhC_2$–BQQDI–$C_7$** |
|---|---|---|---|
| **Formula** | $C_{35}H_{26}N_4O_4$ | $C_{36}H_{28}N_4O_4$ | $C_{37}H_{30}N_4O_4$ |
| **MW** | 566.60 | 580.62 | 594.65 |
| **Temperature (K)** | 298 | 298 | 293 |
| **Crystal System** | Monoclinic | Monoclinic | Monoclinic |
| **Space Group** | *Pn* | *Pn* | *Pn* |
| ***a*** **(Å)** | 7.7195(2) | 7.8191(2) | 7.8265(5) |
| ***b*** **(Å)** | 4.98810(10) | 4.96240(10) | 4.9428(4) |
| ***c*** **(Å)** | 35.0076(10) | 35.6599(11) | 35.626(3) |
| ***α*** **(˚)** | 90 | 90 | 90 |
| ***β*** **(˚)** | 91.243(6) | 92.408(7) | 92.355(7) |
| **γ (˚)** | 90 | 90 | 90 |
| **V ($Å^3$)** | 1347.67(6) | 1382.44(6) | 1377.01(18) |
| ***Z*** | 2 | 2 | 2 |
| **$D_x$ (g $cm^{-3}$)** | 1.396 | 1.395 | 1.434 |
| ***μ*** **(mm)** | 0.753 | 0.747 | 0.763 |

| | | | |
|---|---|---|---|
| **Refined Parameters** | 389 | 397 | 452 |
| **GOF on $F^2$** | 1.196 | 1.013 | 1.593 |
| **R1 [I > 2σ(I)]** | 0.0642 | 0.0643 | 0.1282 |
| **wR2 (all data)** | 0.1888 | 0.1823 | 0.3751 |
| **$\Delta\rho_{min, max}$ (e Å$^{-3}$)** | -0.47, 0.74 | -0.25, -0.25 | -0.56, 0.48 |

Alkyl Chain Conformation

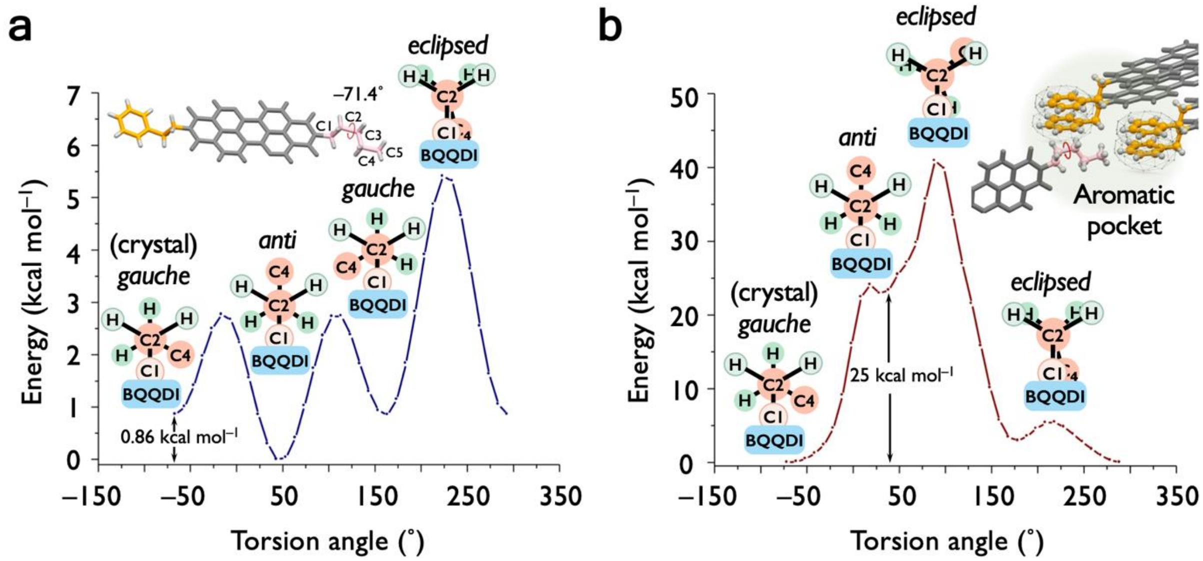


Figure S11. **a** Torsion angle energy scan (C2–C3, starting from –71.4˚) of monomer. **b** Torsion angle energy scan of pentamer (structures from the single crystal) of $PhC_2$–BQQDI–$C_5$ calculated at the B3LYP/6-31+G(d) level of theory.

Temperature-Variant Powder X-Ray Diffractions (PXRD)

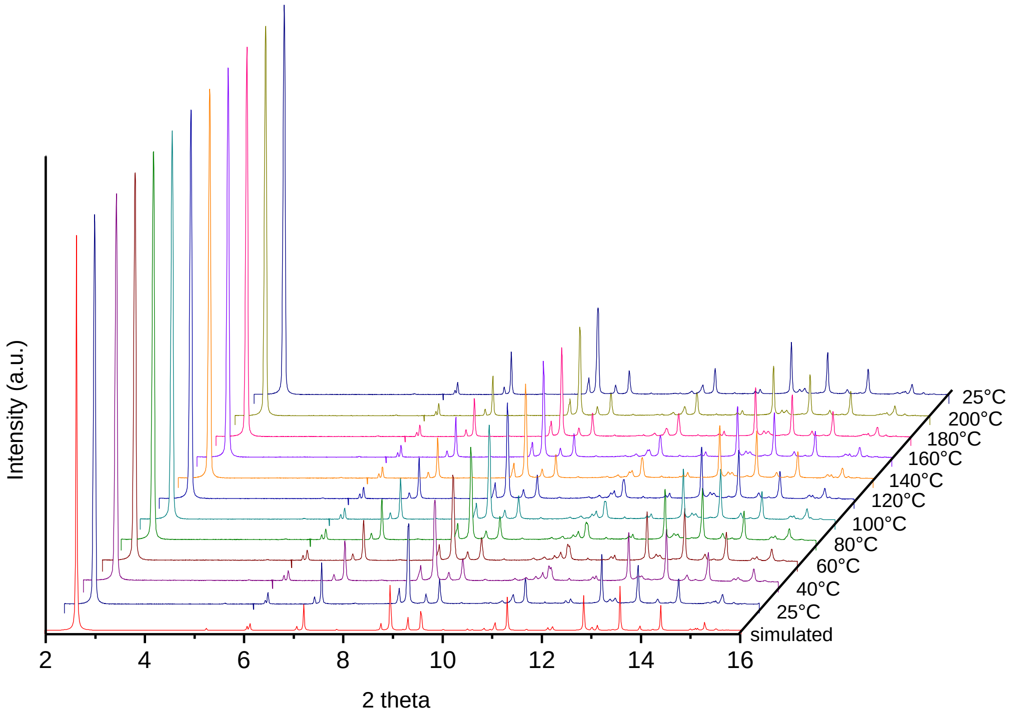


Figure S12. Temperature-variant PXRD plots of $PhC_2$–BQQDI–$C_5$.X-Ray Diffraction of Edge-Casted Thin-Films

The X-ray irradiation was set perpendicular and parallel to the thin film for in-plane and out-of-plane diffractions, respectively.

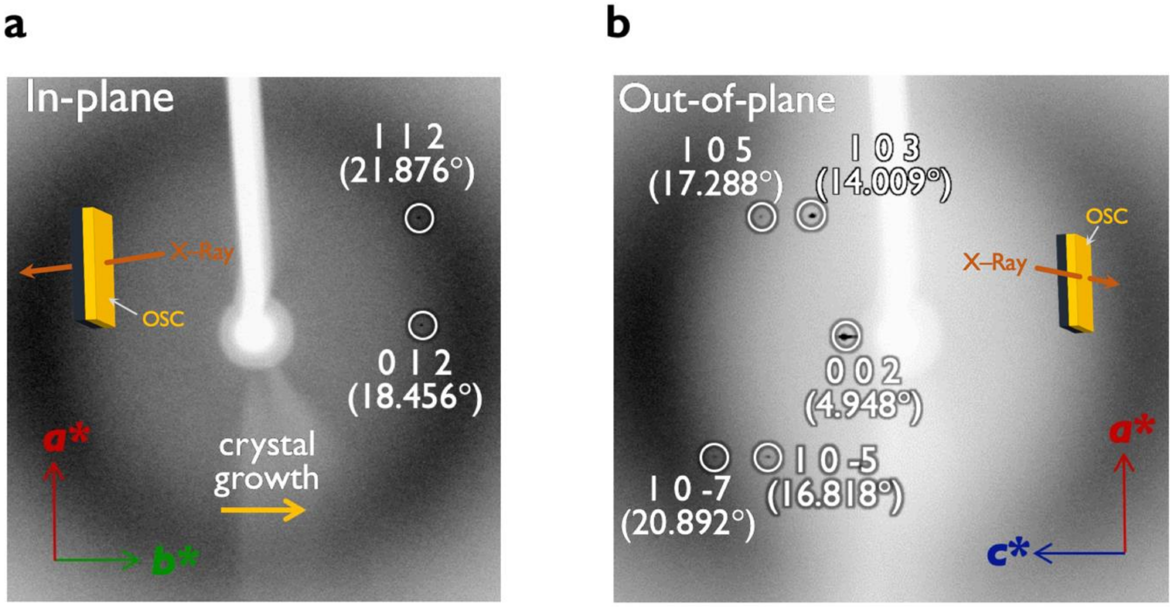


Figure S13. In-plane and out-of-plane thin-film XRD of $PhC_2$–BQQDI–$C_5$.

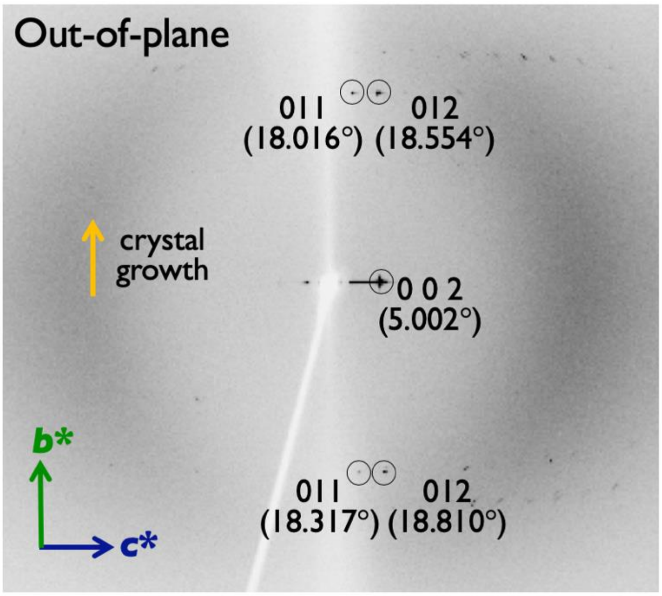


Figure S14. Out-of-plane thin-film XRD of $PhC_2$–BQQDI–$C_6$.

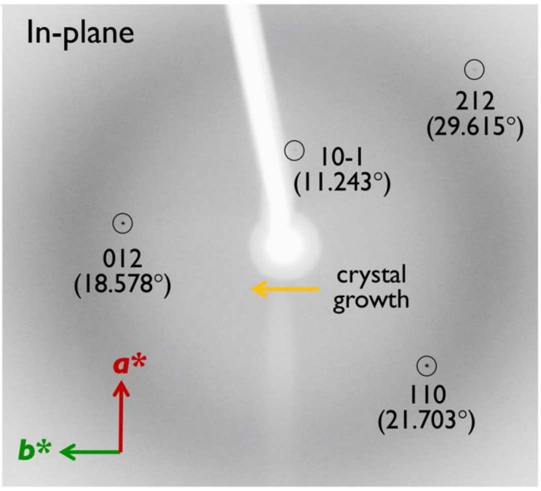


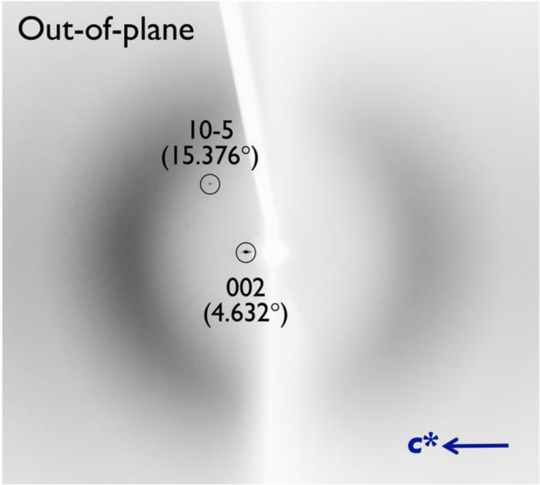


Figure S15. In-plane and out-of-plane thin-film XRD of $PhC_2$–BQQDI–$C_7$.

## 6. Molecular Dynamic Simulations

Molecular dynamics (MD) simulations of single crystal structures in this study were carried out by using the MD program GROMACS 2016.3. Since the intra- and interatomic interactions should be treated explicitly for analyzing the atomistic dynamics, an all-atom model was employed in accordance with generalized Amber force field parameters[64]. The partial atomic charges of the simulated molecules were calculated using the restrained electrostatic potential (RESP)[65] methodology, based on DFT calculations with the 6-31G(d) basis set using the GAUSSIAN 09 program[63].

For each system, the pre-equilibration run was initially performed at the given temperature for 5 ns after the steepest descent energy minimization. All systems were subjected to pre-equilibration runs in the NTV ensemble before their equilibration runs. During the pre-equilibration runs for the NTV ensemble, the Berendsen thermostat[66] was used to maintain the temperature of the system with relaxation time of 0.2 ps and the volume of the MD cell was kept constant. Subsequently, for the NTP ensemble the equilibration run was performed using the Nosé-Hoover thermostat[67–69] and Parrinello-Rahman barostat[70] with relaxation times of 1.0 and 5.0 ps, respectively. For all MD simulations in the NTP ensemble, the pressure of the system was kept at 1.0 bar. The smooth particle-mesh Ewald (PME)[71] method was employed to treat the long-rang electrostatic interactions and the real space cutoff and the grid spacing are 1.2 and 0.30 nm, respectively. The time step was set to 1 fs.

To compare temperature dependence of thermal atomic fluctuations between different molecules, we calculated the B-factors related to the thermal stability as expressed below:

$$B = \frac{8}{3}\pi^2\Delta_i^2$$

where $\Delta_i$ is the root mean square fluctuations (RMSF) of atom $i$. The RMSF values can be estimated by using following equation:

$$\Delta_i = \sqrt{\frac{1}{T}\sum_{j=1}^{T}\left|\boldsymbol{r}_i(t_j) - \bar{\boldsymbol{r}}_i\right|^2}$$

where $T$ is the time step, $\boldsymbol{r}_i(t_j)$ is the position coordinate of atom $i$, and $\bar{\boldsymbol{r}}_i$ is the average of $\boldsymbol{r}_i(t_j)$. The RMSF values were analyzed from MD trajectories during the last 10 ns in the equilibrium.

By using the atomic coordinates at the 100 ns acquired by the MD simulation, transfer integrals t1−3 were calculated over 500 dimers at the PBEPBE/6-31G(d) level of theory.

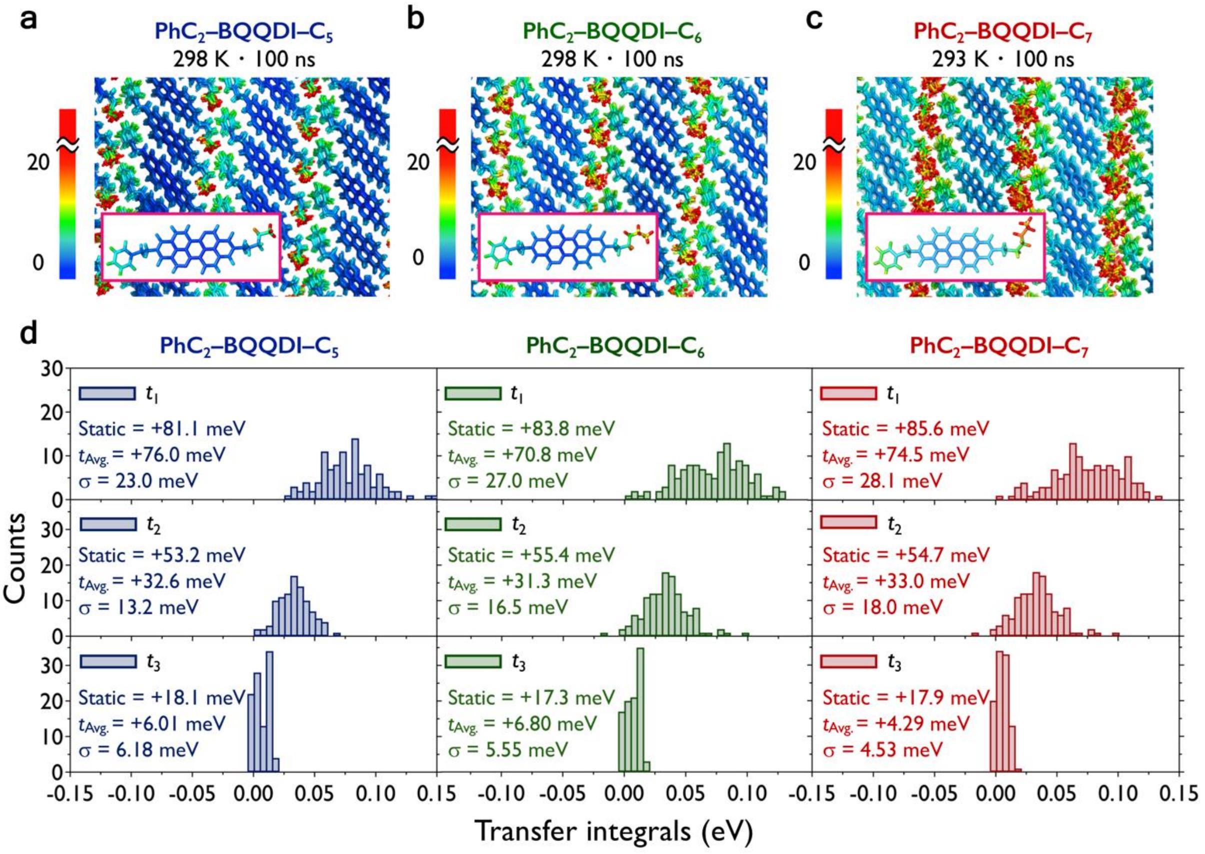


Figure S16. **a–c** Color-coded B-factor (Å$^2$) distribution of $PhC_2$–BQQDI–$C_n$ ($n$= 5, 6, and 7) obtained from the trajectories during the last 10 ns of a 100 ns MD simulations in the NTP ensemble and variant transfer integrals ($t_1$ and $t_3$) at 100 ns of the MD simulations. **d–f** Variant $t$ value distributions and standard deviations (σ) revealing the magnitude of the dynamic fluctuations.Solubility

Table S3 Solubility of $PhC_2$–BQQDI, $C_8$–BQQDI, and $PhC_2$–BQQDI–$C_n$ in $o$–DCB at 100 ˚C.

| **Compound** | **Solubility (wt%)** |
|---|---|
| $PhC_2$–BQQDI | < 0.0007 |
| $C_8$–BQQDI | 0.019 |
| $PhC_2$–BQQDI–$C_5$ | 0.0015 |
| $PhC_2$–BQQDI–$C_6$ | 0.0016 |

| | |
|---|---|
| $PhC_2$–BQQDI–$C_7$ | 0.0023 |

## 7. OFET Fabrications

Preparation of OFET Substrates

The highly $n^{++}$-doped silicon wafer with thermally grown $SiO_2$ layer was used as the substrate. Before surface functionalization, the silicon wafer was cleaned by UV−$O_3$ treatment. Here two polymeric functionalization materials were attempted. One is a fluorine-containing cross-linked polymer, AL-X601 (supplied by AGC Inc.), which was diluted with propylene glycol monomethyl ether acetate and spin-coated onto $SiO_2$ (200 nm), followed by curing at 180˚C for 10 min in air. The thickness of AL-X601 was 55 nm. Another is 13 nm-thick parylene (diX-SR, KISCO Ltd.) deposited onto $SiO_2$ (100 nm) by chemical vapor deposition.

Fabrications of Solution-Processed Single-Crystalline Thin Films

$PhC_2$–BQQDI–$C_n$ were investigated in the bottom-gate, top-contact OFET structure. Preparations of single-crystalline thin films were carried out by the solution-processed edge-casting method[72]. Thin-film crystals of $PhC_2$–BQQDI–$C_n$ were grown from 0.02–0.03 wt% 1-methylnaphthalene solutions at 90–115 ˚C. After the completion of crystallization, thin films were thoroughly dried in a vacuum oven at 100 °C for 10 hours. Then, 40 nm-thick gold layers were vacuum deposited through a metal shadow mask, acting as source and drain electrodes. Objective channel regions were edged by the conventional Nd:YAG laser etching technique or manually by using cotton swabs. Before measurements, thermal annealing at 100 ˚C for 10 hours were conducted to remove residual water and improve gold electrode−semiconductor contacts.

Fabrication of Large-Area Single-Crystalline Thin Films

The single-crystalline film of $PhC_2$–BQQDI–$C_5$ (0.02 wt% in 1-methylnaphthalene) was prepared on a glass substrate encapsulated by a 55 nm-thick AL-X601 insulating layer by means of the continuous edge-casting method. The stage temperature was maintained at 140 ˚C and the velocity of the moving stage was set to 24 µm $s^{-1}$.OFET Measurements

Electrical evaluations of the TFTs were conducted on a Keithley 4200-SCS semiconductor parameter analyzer in air. For the $SiO_2$/AL-X601 gate dielectrics, the gate capacitance per unit area ($C_i$) was estimated on metal−insulator−metal structures using the semiconductor

parameter analyzer (12.6 nF $cm^{-2}$). Similarly, $C_i$ of the $SiO_2$/diX-SR gate dielectrics was measured to be 26.6 nF $cm^{-2}$. Electron mobility and threshold voltage were extracted from the transfer characteristics by using the conventional equation for the saturation regime:

$$\sqrt{|I_D|} = \sqrt{\frac{W\mu C_i}{2L}}(V_G - V_{th}),$$

where $I_D$ is the drain current, $W$ the channel width, $\mu$ the electron mobility, $C_i$ the gate capacitance per unit area, $L$ the channel length, $V_G$ the gate voltage, and $V_{th}$ the threshold voltage. Thermal stress was applied by annealing the OFETs under vacuum for 10 min, and the measurements were performed in air.

## 8. OFET Performance Evaluations

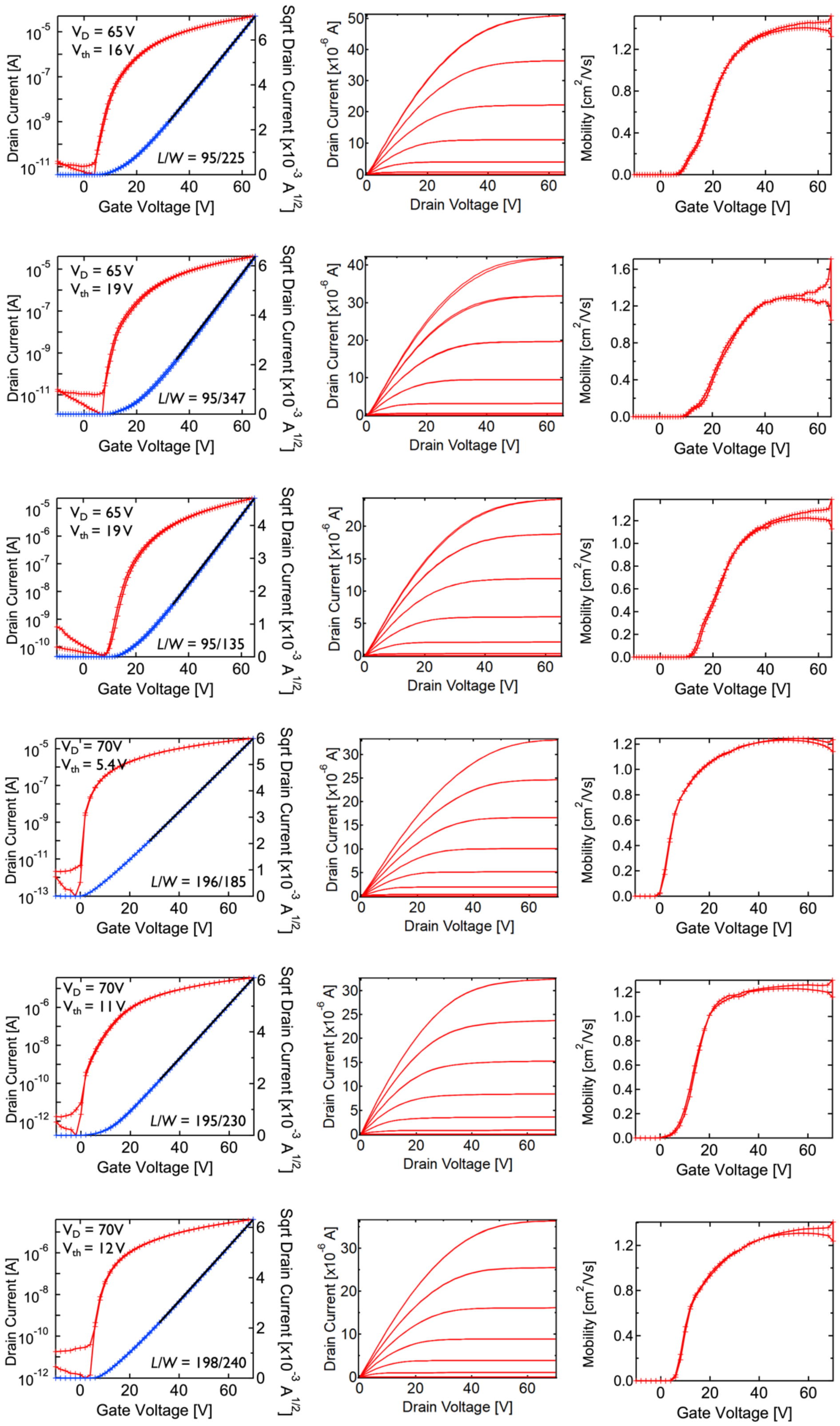


Figure S17. Typical transfer characteristics, output curves, and gate voltage-dependent $\mu_e$ of $PhC_2$–BQQDI–$C_5$ in six different devices.

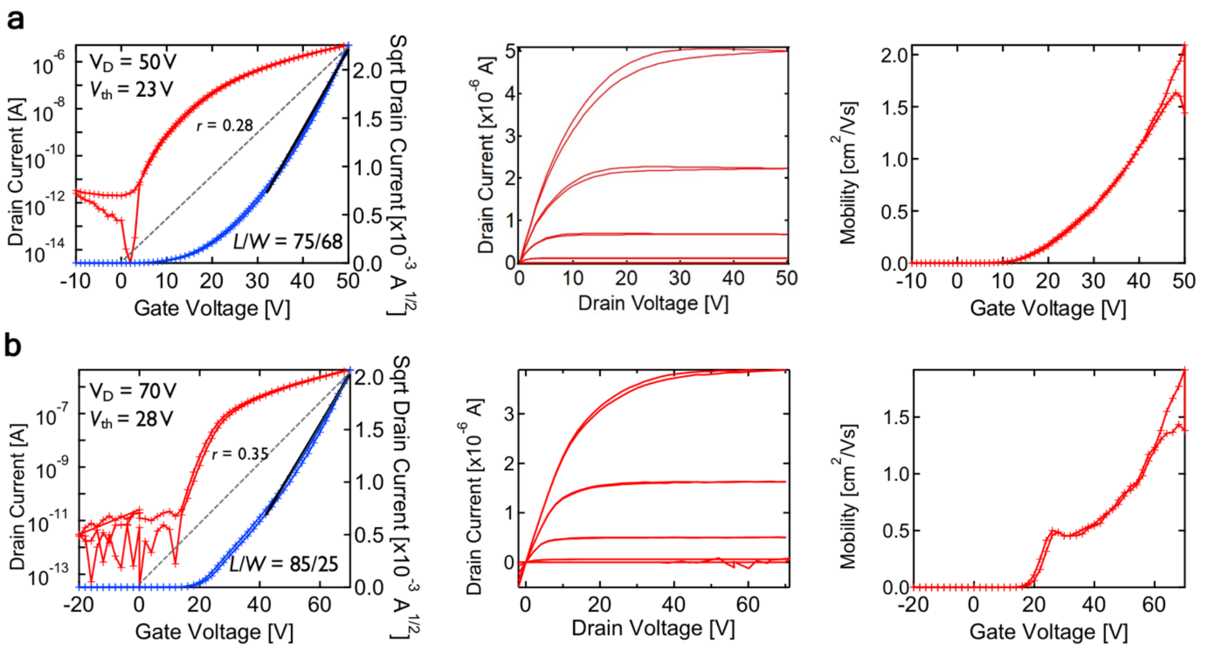


Figure S18. Transfer characteristics, output curves, and gate voltage-dependent $\mu_e$ of **a** $PhC_2$–BQQDI–$C_6$, and **b** $PhC_2$–BQQDI–$C_7$.

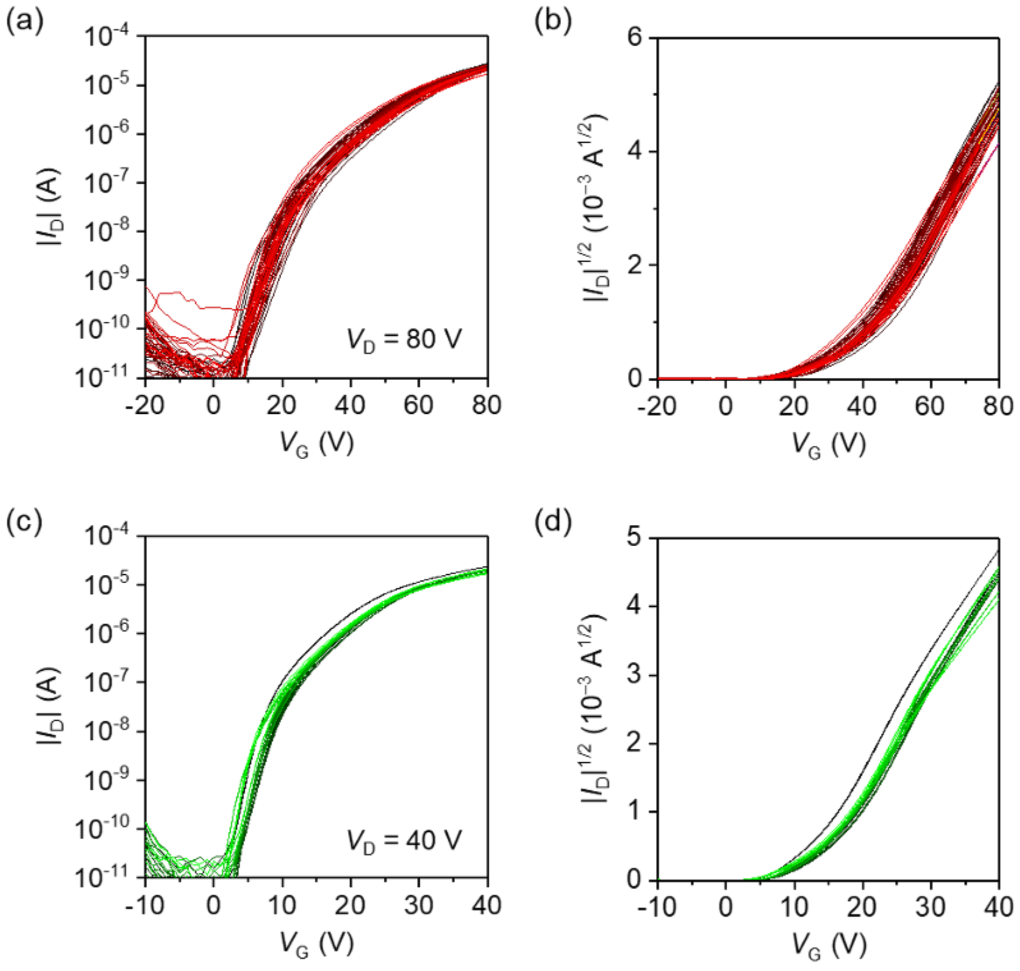

Figure S19. Variation of single-crystalline OFETs based on $PhC_2$−BQQDI−$C_5$ with the channel dimensions $W/L$ = 100 μm/100 μm. **a, b** 24 OFETs with AL-X 601 gate dielectric. **c, d** 12 OFETs with diX-SR gate dielectric.

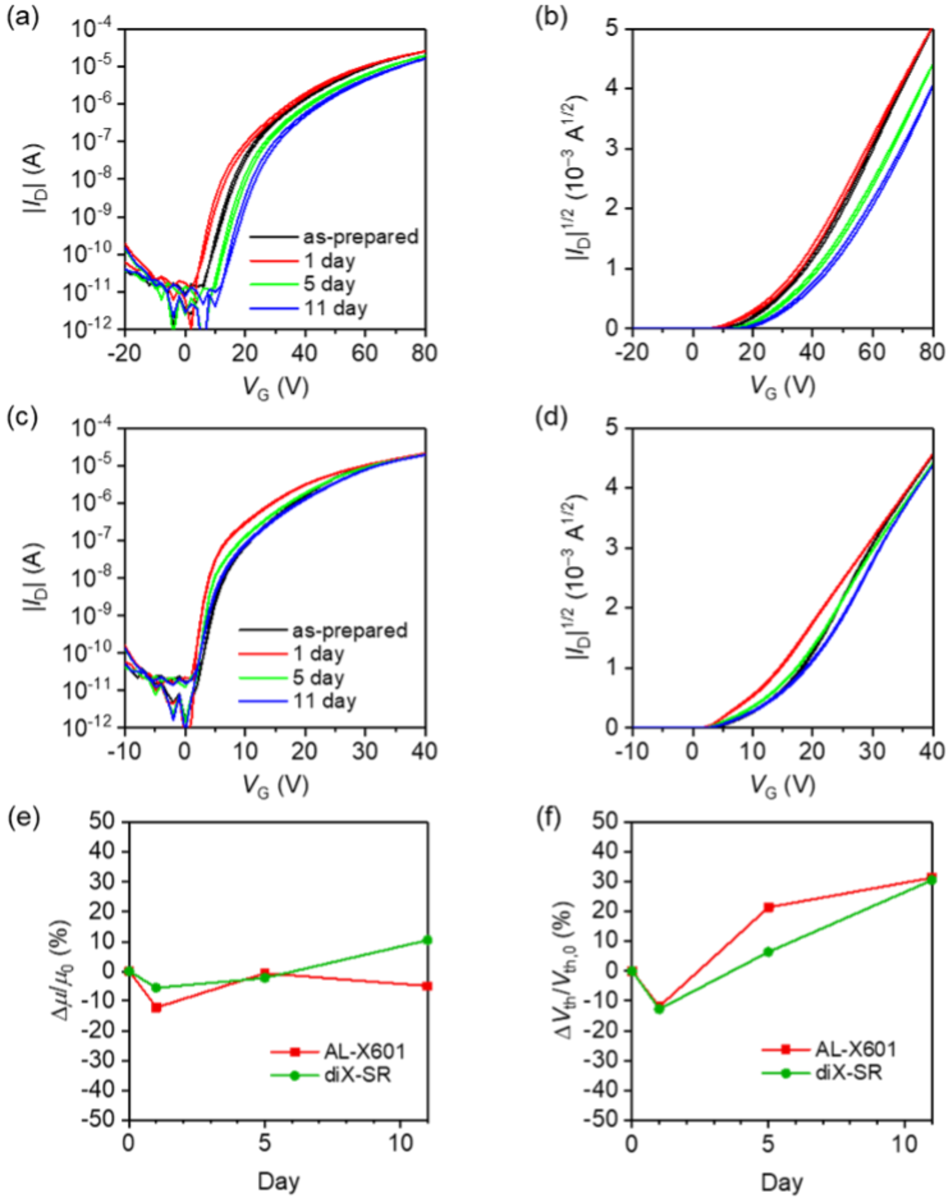


Figure S20. Shelf-life stability of single-crystalline OFETs based on $PhC_2$−BQQDI−$C_5$ under ambient atmosphere. Transfer characteristics with **a, b** AL-X601 and **c, d** diX-SR gate dielectrics. Traces of **e** the normalized apparent $\mu$ and **f** the normalized $V_{th}$. The initial $\mu$ and

$V_{th}$ ($\mu_0$ and $V_{th,0}$, respectively) are 1.61 cm$^2$ V$^{-1}$ s$^{-1}$ and 29.6 V for AL-X601 and 1.57 cm$^2$ V$^{-1}$ s$^{-1}$ and 8.5 V for diX-SR, respectively. $W/L$ = 100 μm/100 μm.

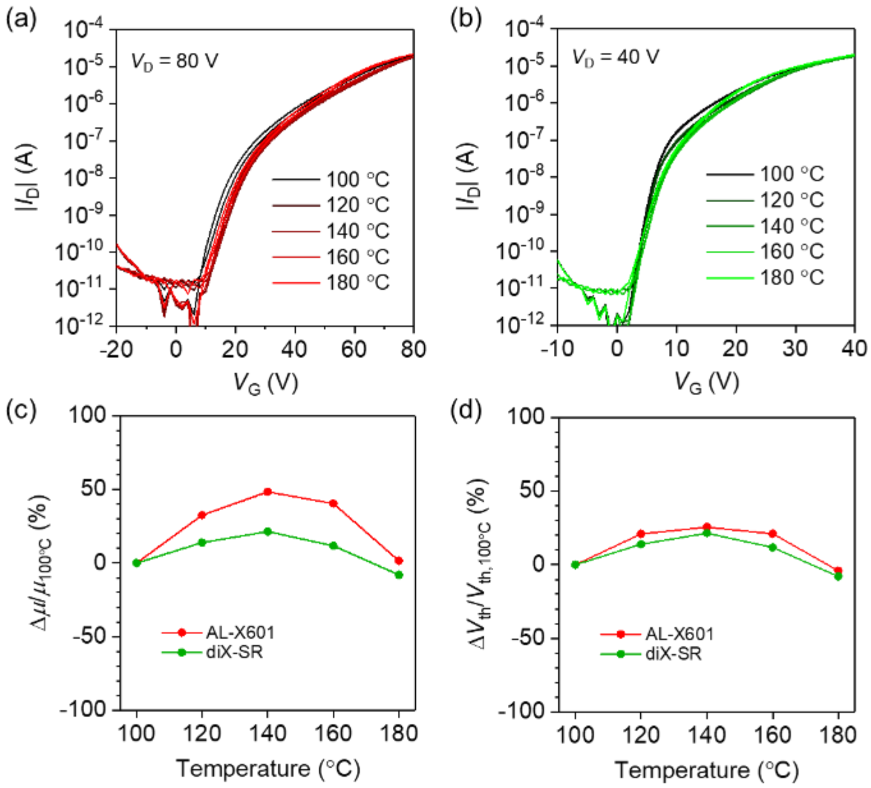


Figure S21. Thermal stress durability of single-crystalline OFETs based on $PhC_2$–BQQDI–$C_5$. Transfer curves of OFETs with **a** AL-X601 and **b** diX-SR gate dielectrics after annealing for 10 min at each temperature. Stress temperature dependence of the change in **c** $\mu$ and **d** $V_{th}$ with respect to the values at 100 °C stress. The $\mu$ and $V_{th}$ at 100 °C stress ($\mu_{100°C}$ and $V_{th,100°C}$, respectively) are 1.78 cm$^2$ V$^{-1}$ s$^{-1}$ and 37.1 V for AL-X601 and 1.57 cm$^2$ V$^{-1}$ s$^{-1}$ and 9.8 V for diX-SR, respectively. $W/L$ = 100 μm/100 μm.

Table S4. Summary of OFET performances of current asymmetric $PhC_2$–BQQDI–$C_n$ derivatives and the previously reported $PhC_2$–BQQDI.

| Compound | Maximum $\mu$ ($cm^2\ V^{-1}\ s^{-1}$) | $V_{th}$ (V) | $r$ |
|---|---|---|---|
| $PhC_2$–BQQDI–$C_5$ | 1.90$^a$, 1.83$^b$ | 35$^a$, 11$^b$ | 0.32$^a$, 0.53$^b$ |
| $PhC_2$–BQQDI–$C_6$ | 1.2 | 23 | 0.26 |
| $PhC_2$–BQQDI–$C_7$ | 1.0 | 28 | 0.35 |
| $PhC_2$–BQQDI | 3.0 | 4.0 | 0.89 |

$^a$Device measured on AL-X601. $^b$Device measured on diX-SR. Note that $V_{th}$ of $PhC_2$–BQQDI–$C_5$ is strongly depended on gate capacitance.

## 9. Single-Crystalline Thin Films

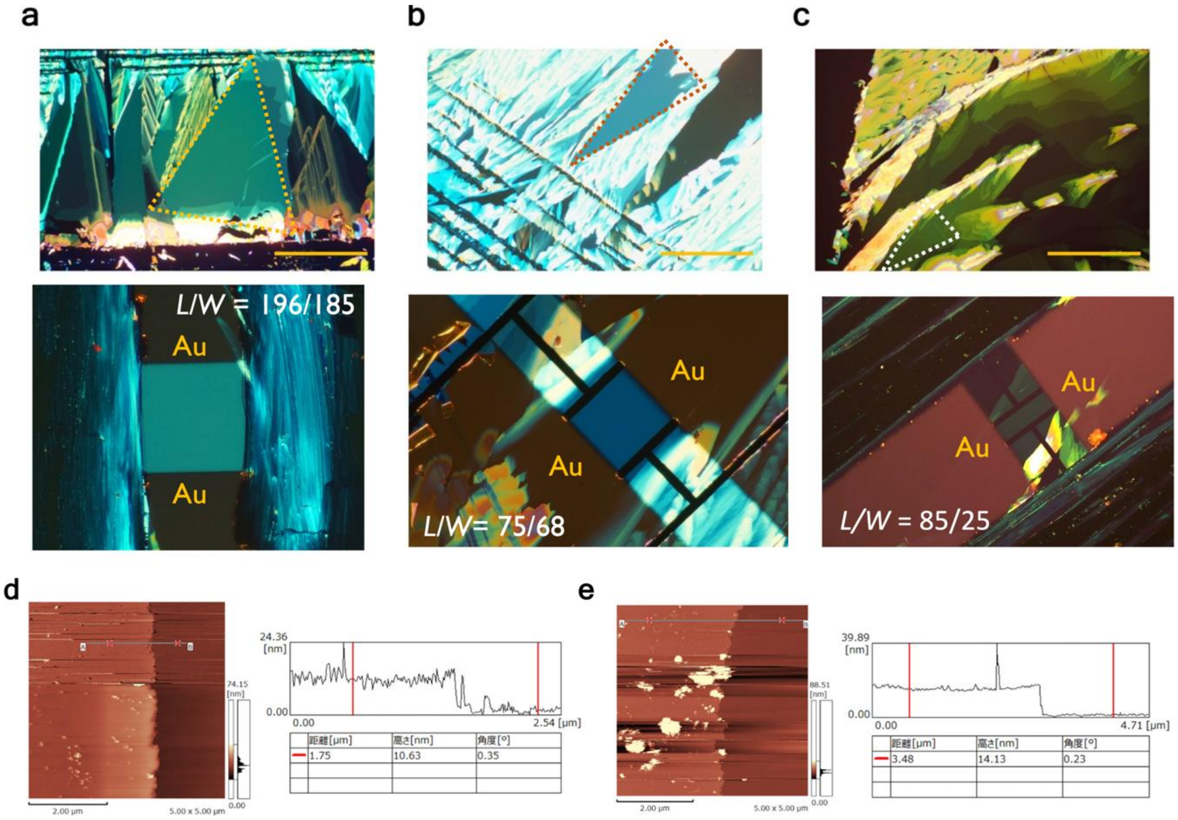


Figure S22. Polarized optical microscopic images of single-crystalline thin films of **a** $PhC_2$–BQQDI–$C_5$, **b** $PhC_2$–BQQDI–$C_6$, and **c** $PhC_2$–BQQDI–$C_7$, fabricated at the best temperatures of 115˚C, 110˚C, and 90˚C, respectively (scale bar = 0.5 mm). The dotted triangles indicate the single-crystalline regions (with similar film thicknesses) for device fabrications. Crystal thickness measured by AFM for **d** $PhC_2$–BQQDI–$C_5$, **e** $PhC_2$–BQQDI–$C_6$.

## 12. Large-Area Single-Crystalline Thin Film

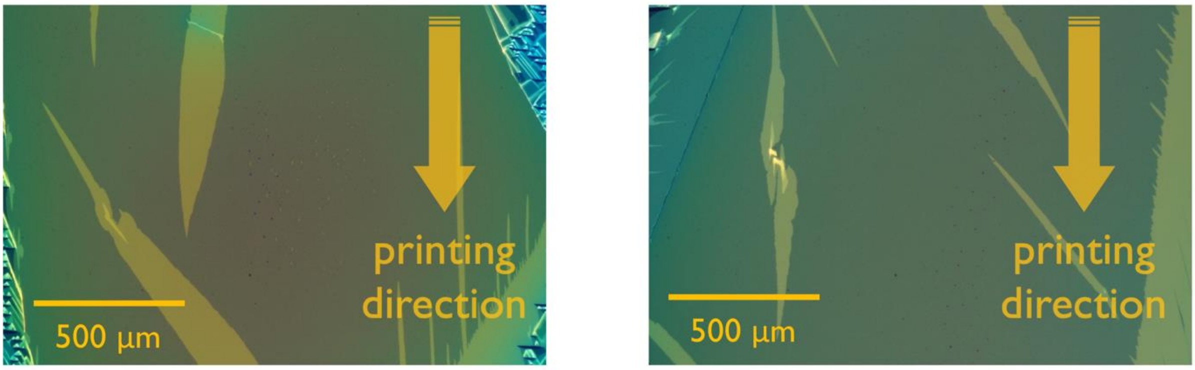


Figure S23. Polarized optical microscopic images of large-area single-crystalline thin films of $PhC_2$–BQQDI–$C_5$.

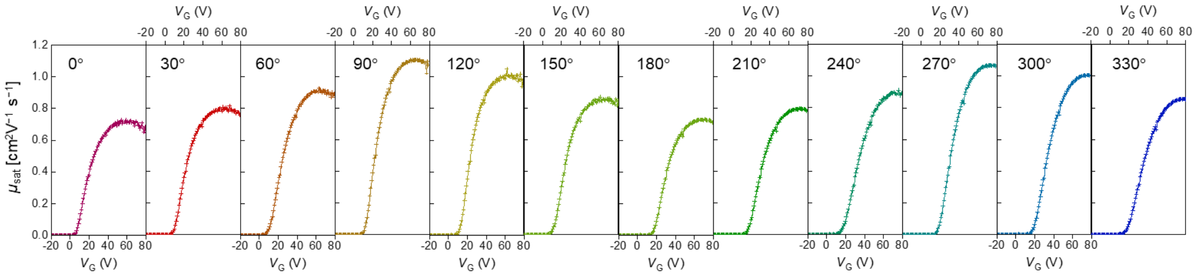


Figure S24. $V_G$ dependent saturated $\mu_e$ ($\mu_{sat}$) of large-area single-crystalline thin film of $PhC_2$–BQQDI–$C_5$ with their corresponding angle (°) relative to the printing direction.

## 13. Effective Masses of Different Molecular Layers

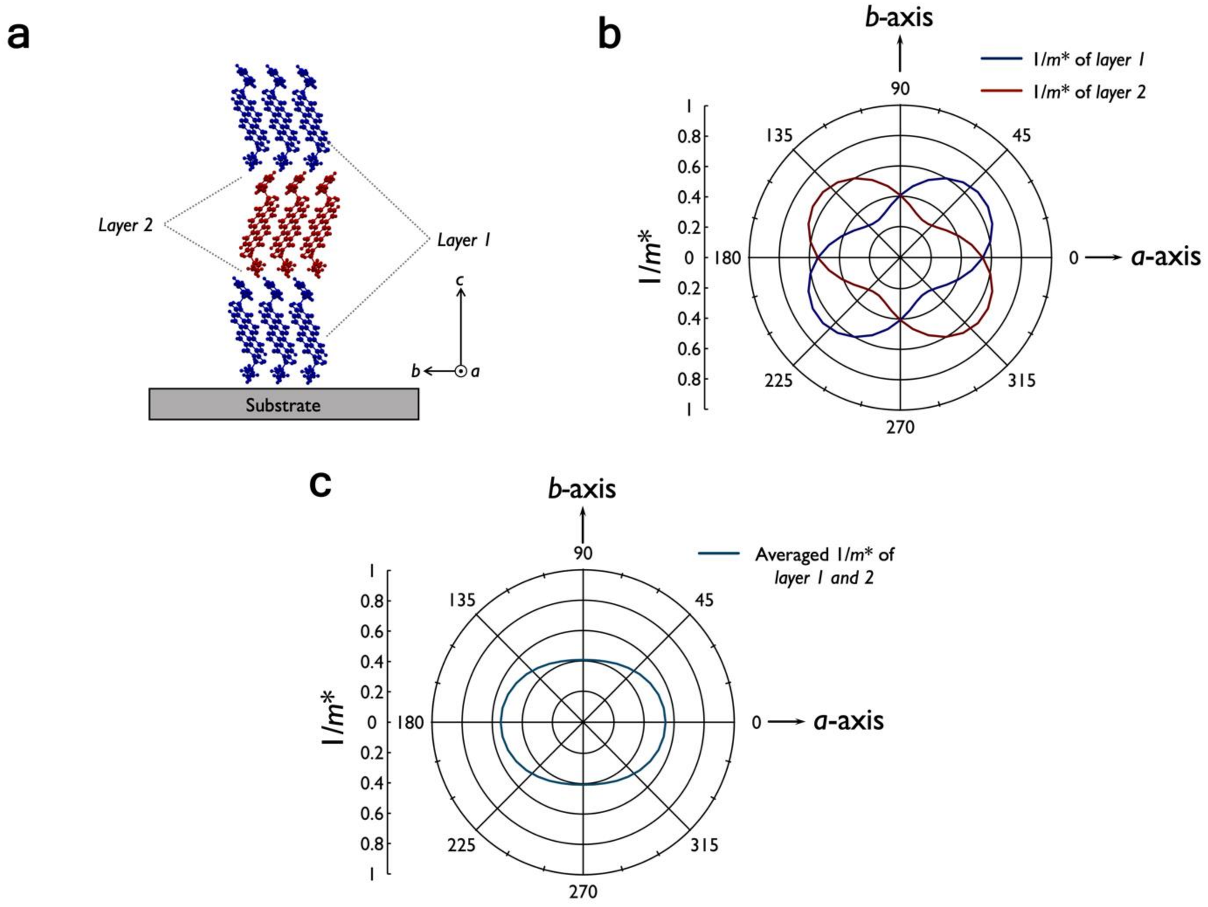


Figure S25. **a** Two different molecular layers in the single-crystal structure of $PhC_2$–BQQDI–$C_5$ (indicated by different colors). **b** The corresponding angle-dependent inversed effective masses. **c** Averaged angle-dependent inversed effective masses from layers 1 and 2.

## 14. References


(1) Yamagiwa, N.; Okabe, T.; Suto, Y.; Iwasaki, G. Acidic Solvent-Free Removal of Amine-Protecting Diphenylmethyl Groups in the Presence of Camphorsulfonic Acid. *Chem. Lett.* **2017**, *46*, 1456–1458.

(2) Schmidt, M. W.; Baldridge, K. K.; Boatz, J. A.; Elbert, S. T.; Gordon, M. S.; Jensen, J. H.; Koseki, S.; Matsunaga, N.; Nguyen, K. A.; Su, S.; Windus, T. L.; Dupuis, M.; Montgomery, J. A. General Atomic and Molecular Electronic Structure System. *J. Comput. Chem.* **1993**, *14*, 1347–1363.

(3) Coropceanu, V.; Cornil, J.; da Silva Filho, D. A.; Olivier, Y.; Silbey, R.; Brédas, J. L. Charge Transport in Organic Semiconductors. *Chem. Rev.* **2007**, *107*, 926–952.

(4) Boys, S. F.; Bernardi, F. The Calculation of Small Molecular Interactions by the Differences of Separate Total Energies. Some Procedures with Reduced Errors. *Mol. Phys.* **1970**, *19*, 553–566.

(5) Frisch, M. J. et al. Gaussian 09, Revision A.02. *Gaussian 09, Revision A.02*. 2009.

(6) Kato, K.; Tanaka, H. Visualizing Charge Densities and Electrostatic Potentials in Materials by Synchrotron X-Ray Powder Diffraction. *Adv. Phys. X* **2016**, *1*, 55–80.

(7) Kato, K.; Tanaka, Y.; Yamauchi, M.; Ohara, K.; Hatsui, T. A Statistical Approach to Correct X-Ray Response Non-Uniformity in Microstrip Detectors for High-Accuracy and High-Resolution Total-Scattering Measurements. *J. Synchrotron Radiat.* **2019**, *26*, 762–773.

(8) Uemura, T.; Hirose, Y.; Uno, M.; Takimiya, K.; Takeya, J. Very High Mobility in Solution-Processed Organic Thin-Film Transistors of Highly Ordered [1]Benzothieno[3,2-b]Benzothiophene Derivatives. *Appl. Phys. Express* **2009**, *2*, No. 111501.

(9) Wang, J.; Wolf, R. M.; Caldwell, J. W.; Kollman, P. A.; Case, D. A. Development and Testing of a General Amber Force Field. *J. Comput. Chem.* **2004**, *25*, 1157–1174.

(10) Bayly, C. I.; Cieplak, P.; Cornell, W. D.; Kollman, P. A. A Well-Behaved Electrostatic Potential Based Method Using Charge Restraints for Deriving Atomic Charges: The RESP Model. *J Phys. Chem.* **1993**, *97*, 10269–10280.

(11) Berendsen, H. J. C.; Postma, J. P. M.; Van Gunsteren, W. F.; Dinola, A.; Haak, J. R. Molecular Dynamics with Coupling to an External Bath. *J. Chem. Phys.* **1984**, *81*, 3684–3690.

(12) Nosé, S. A Unified Formulation of the Constant Temperature Molecular Dynamics Methods. *J. Chem. Phys.* **1984**, *81*, 511–519.

(13) Nosé, S. A Molecular Dynamics Method for Simulations in the Canonical Ensemble. *Mol. Phys.* **1984**, *52*, 255–268.

(14) Hoover, W. G. Canonical Dynamics: Equilibrium Phase-Space Distributions. *Phys. Rev. A* **1985**, *31*, 1695–1697.

(15) Parrinello, M.; Rahman, A. Polymorphic Transitions in Single Crystals: A New Molecular Dynamics Method. *J. Appl. Phys.* **1981**, *52*, 7182–7190.

(16) Darden, T.; York, D.; Pedersen, L. Particle Mesh Ewald: An N·log(N) Method for Ewald Sums in Large Systems. *J. Chem. Phys.* **1993**, *98*, 10089–10092.
